# Isolation, phylogenetics, and characterization of a new PDCoV strain that affects cellular gene expression in human cells

**DOI:** 10.3389/fmicb.2025.1534907

**Published:** 2025-03-26

**Authors:** Xiaozhu Yang, Hanwei Yin, Mengyuan Liu, Xuemei Wang, Tao Song, Aiai Song, Yibo Xi, Ting Zhang, Zilong Sun, Wei Li, Sheng Niu, Farwa Zainab, Chenyang Wang, Ding Zhang, Haidong Wang, Bo Yang

**Affiliations:** ^1^College of Veterinary Medicine, Shanxi Agricultural University, Jinzhong, China; ^2^College of Animal Science and Technology, Hebei Normal University of Science and Technology, Qinhuangdao, China; ^3^Xianyang Regional Wen's Animal Husbandry Co., Ltd., Xianyang, China; ^4^School of Management Shanxi Medical University, Taiyuan, China

**Keywords:** PDCoV, Huh7 cells, phylogenetic tree, transcriptome analysis, immune response

## Abstract

**Introduction:**

Porcine deltacoronavirus (PDCoV) is an enteropathogenic coronavirus that causes acute diarrhea, vomiting, dehydration, and even death in piglets, resulting in serious economic losses to the pork industry worldwide. PDCoV has received much attention owing to its broad host range, including humans, posing a potential threat to public health. However, the prevalence, characteristics, and host cellular gene expression of PDCoV remain poorly understood.

**Methods:**

In this study, a new PDCoV strain (CHN/SX-Y/2023, GenBank number PQ373831) was successfully isolated, identified, and subjected to phylogenetic tree and transcriptome analysis in human hepatoma (Huh7) cells following PDCoV infection.

**Results:**

The results showed that the CHN/SX-Y/2023 strain belongs to the Chinese lineage and causes cytopathic effects in canonical cell lines (LLC-PK1 and ST cells) and other cell lines (Huh7 and LMH cells). However, HEK-293T, EEC, MDBK, and Vero-CCL81 cells were not found to be susceptible in this study. Based on transcriptome analysis, 1,799 differentially expressed genes (DEGs) were upregulated and 771 were downregulated during PDCoV infection.

**Discussion:**

Among the upregulated genes, *FCGR1A*, *VSIG1*, *TNFRSF9*, and *PLCXD3* are associated with immunity, inflammation, and lipid catabolism. Moreover, Kyoto Encyclopedia of Genes and Genomes analysis revealed that the upregulated DEGs were significantly enriched in the MAPK, TNF, and NF-κB signaling pathways and viral protein interactions with cytokines and cytokine receptors. Protein–protein interaction networks showed that the upregulated genes *CXCL8*, *DUSP1*, *PTGS2*, and *IL15* were associated with inflammation and immunity. In addition, the protein levels of p-IRF3, LC3-II, and ACSL4 increased, suggesting that PDCoV infection in Huh7 cells induces an intrinsic immune response, cellular autophagy, and ferroptosis. Collectively, our findings provide new insights into the characteristics and mechanisms of PDCoV infection.

## 1 Introduction

Porcine deltacoronavirus (PDCoV), also referred to as coronavirus HKU15, is a member of the genus *Deltacoronavirus* of the family Coronaviridae (Kikuti et al., 2024). PDCoV causes diarrhea, dehydration, vomiting, and enteric damage in neonatal piglets, similar to those caused by porcine epidemic diarrhea virus (PEDV) and transmissible gastroenteritis virus (TGEV) (Bahoussi et al., 2022; Yin et al., 2022). PDCoV was first reported in pigs in Hong Kong (Woo et al., 2012), and received significant attention after an outbreak in Ohio, United States in 2014 (Wang et al., 2014; Shan et al., 2024). Interestingly, a previous study reported that the PDCoV (CHN/AH-2004) strain was isolated from Anhui Province, China, as early as 2004 (Dong et al., 2015). PDCoV has spread rapidly throughout the United States, China, South Korea, Japan, Thailand, and Vietnam, posing an enormous threat and economic loss to the commercial pork industry (Zhao et al., 2019; Kong et al., 2022).

PDCoV is an enveloped, single-stranded, positive-sense RNA virus that is pleomorphic, with a diameter of 60–180 nm (Ma et al., 2015). The genome of PDCoV is appropriately 25.4 kb in length, and encodes 15 mature nonstructural proteins, four structural proteins and three accessory nonstructural proteins (Tang et al., 2021). Among the structural proteins, S plays an important role in the binding of the virus to host receptors (Liu et al., 2022). The E and M transmembrane proteins are involved in envelope formation and viral release (Masters, 2006; Schoeman and Fielding, 2019). The PDCoV N protein is highly conserved and binds to the viral RNA (Lee and Lee, 2015). Recent studies have shown that PDCoV uses aminopeptidase N (APN) from different species to enter host cells and exhibits a broad spectrum of infectivity (Li et al., 2018; Yang et al., 2021). Notably, PDCoV strains have been isolated from blood samples of Haitian children (Lednicky et al., 2021). Furthermore, human hepatoma (Huh7) and HeLa cells are susceptible to PDCoV, while human lung carcinoma cells (A549) support PDCoV replication in the presence of trypsin (Fang et al., 2021). These studies indicate that PDCoV poses a potential risk of human infection, thereby posing a threat to public health (Li et al., 2022; Alhamo et al., 2022).

In this study, we explore phylogenetics, infected characterization, and transcriptome analysis of a new PDCoV strain, which are crucial to provide information for the host response to PDCoV infection. Our data enrich understanding of the epidemiology and pathogenesis of PDCoV strains and provide important insights into their prevention and control.

## 2 Materials and methods

### 2.1 Cells and main reagents

LLC Porcine Kidney Epithelial (LLC-PK1), Swine Testis (ST), Baby Hamster Syrian Kidney-21 (BHK-21), African Green Monkey Kidney (Vero-CCL81), Human Embryonic Kidney 293 cells stably expressing the SV40 large T antigen (HEK-293T), Madin-Darby Canine Kidney (MDCK), Goat enteroendocrine cells (EEC), Leghorn Male Hepatoma (LMH) cells, and Huh7 cells were cultured in Dulbecco’s modified Eagle medium (DMEM) with high glucose (Gibco, United States). Madin-Darby bovine kidney (MDBK) cells were grown in RPMI 1640 medium (Gibco, United States). Ten percent fetal bovine serum (FBS, Gibco, United States) and 1% penicillin–streptomycin solution (Gibco, United States) were added to the medium. The cells were cultured at 37°C containing 5% CO_2_. The cell information is shown in Supplementary Table S1. The cell culture conditions used to infect different cells with PDCoV were as follows: washing of cells (LLC-PK1, ST, Vero-CCL81, MDCK, EEC, LMH, and Huh7) with PBS two times, virus incubation for 2 h in fresh DMEM containing 10 μg/mL trypsin (Sigma, United States), in fresh DMEM containing 5 μg/mL trypsin (Sigma, United States) (in HEK-293T and BHK-21), and in fresh RPMI 1640 containing 10 μg/mL trypsin (Sigma, United States) (in MDBK). The mouse anti-PDCoV N monoclonal antibody was preserved in our laboratory. GAPDH antibody was purchased from Proteintech (Wuhan, China). Goat anti-Mouse IgG (H + L) Highly Cross-Adsorbed Secondary Antibody, and Alexa Fluor^™^ Plus 594 were purchased from Thermo Fisher Scientific (China).

### 2.2 Clinical samples

Clinical intestinal samples were collected from Xianyang Regional Wen’s Animal Husbandry Co., Ltd. in 2023. After three freeze–thaw cycles, the samples were homogenized, vortexed, and centrifuged. The supernatants were then filtered through a 0.22 μm sterile filter and stored at −80°C.

### 2.3 Virus isolation and electron microscopic observations

PDCoV was isolated from LLC-PK1 cells cultured in T75 flasks. Briefly, after incubation of the filtered sample with 10 mL DMEM and 10 μg/mL trypsin (Sigma, United States) for 2 h, the cells were washed and cultured in DMEM supplemented with 10% FBS and 1% penicillin–streptomycin solution at 37°C in a 5% CO_2_ incubator. When an obvious cytopathic effect (CPE) was observed in approximately 90% of the cell monolayers, the T75 flasks were frozen and thawed three times at −80°C. The supernatants and cells were then harvested and stored at −80°C.

The PDCoV-infected LLC-PK1 cell culture medium was clarified by centrifugation. After filtration through 0.45 μm filters, the PDCoV medium was ultracentrifuged (Beckman Coulter, United States). The prepared samples were stained with an equal volume of 3% phosphotungstic acid in 0.4% sucrose and applied to a 300-mesh Formvar and carbon-coated copper grid. After blotting and drying, the PDCoV grid was examined under a Talos L120C electron microscope (Thermo Fisher Scientific).

### 2.4 TCID_50_ assay

Briefly, viral titers were measured using 50% tissue culture infectious dose (TCID_50_) assays in LLC-PK1 cells in 96-well plates. The cells were washed and PDCoV was inoculated in10-fold serial dilutions in 100 μL DMEM with 10 μg/mL trypsin. Next, the cells were washed, 200 μL of DMEM with 10% FBS and 1% penicillin–streptomycin solution was added after 2 h. CPE was observed for 3–5 days and analyzed using Reed–Muench method.

### 2.5 Western blotting assay

PDCoV at a multiplicity of infection of 1 was adsorbed onto cells in DMEM containing 10 μg/mL trypsin for 2 h. The cells were washed and DMEM with 10% FBS and 1% penicillin–streptomycin solution was added. The cells were collected following PDCoV infection. The protein extracts were prepared from cells by suspension in lysis buffer containing protease inhibitor phenylmethylsulfonyl fluoride (Solarbio, Beijing) for 30 min on ice. The proteins were separated on 10% sodium dodecyl sulfate-polyacrylamide gel electrophoresis gels and transferred to 0.22 μm polyvinylidene difluoride membranes (Immobilon^®^, Merck, China). The membranes were blocked with 10% nonfat milk and incubated overnight with PDCoV N antibodies. After washing three times, the membranes were incubated with a 1:10000 dilution of horseradish peroxidase-labeled goat anti-mouse IgG (H + L) secondary antibody (EASYBIO, Beijing) for 45 min. Protein bands were detected using Pierce ECL Western Blotting Substrate (Thermo Fisher Scientific), and the band density was quantified using ImageJ software (Version 1.38).

### 2.6 Immunofluorescence assay

Briefly, PDCoV-infected LLC-PK1 cells were fixed with 4% paraformaldehyde (Solarbio, Beijing) for 30 min, washed three times, and permeabilized with 0.1% Triton X-100 (Sigma-Aldrich) for 10 min. The fixed cells were blocked with 10% (w/v) skim milk for 1 h and incubated overnight with anti-PDCoV N antibody. The cells were then washed and incubated with Goat Anti-Mouse IgG (H + L) Highly Cross-Adsorbed Secondary Antibody (Alexa Fluor^™^ Plus 594) for 1 h. Nuclei were visualized using 4′,6-diamidino-2-phenylindole nuclear counterstaining (Sigma-Aldrich^®^, Merck, China). Cell observation and imaging were performed using a fluorescence microscope (Leica, Germany).

### 2.7 Whole-genome amplification and sequencing

PDCoV-infected samples were centrifuged and TRIzol reagent (Invitrogen) was added. The RNA samples were stored at −80°C. Reverse transcription was performed using the PrimeScript 1st Strand cDNA Synthesis Kit (Takara). The cDNA was amplified by polymerase chain reaction (PCR) using Taq DNA Polymerase (Takara). Nineteen overlapping primer pairs were designed to amplify the complete PDCoV gene sequence (Supplementary Table S2). The primers and positive recombinant plasmids were purchased from Beijing Tsingke Biotech Co., Ltd. (Beijing, China). The raw genomic sequence fragments were imported into SeqMan in DNASTAR for assembly and annotation. Sequence alignment analysis was performed using ClustalW. The PDCoV sequences obtained in this study have been submitted to GenBank under accession number PQ373831.2.

### 2.8 Phylogenetic and amino acid sequence analysis

All available full-length PDCoV genome nucleotide sequences retrieved from the National Center for Biotechnology Information GenBank database were collected and analyzed. Sequences that were (a) unverified, (b) truncated, or (c) laboratory hosts were excluded. A dataset of 154 PDCoV strains is shown in Supplementary Table S3, aligned by MUSCLE using the maximum likelihood phylogenetic test in Mega-X software (Version 10.1.18). Datasets, annotations, and interactive trees were created using the Interactive Tree of Life and Adobe Illustrator 2020. Amino acid sequences were aligned using ClustalW with MegAlign and JalView software (Version 2.11.4.1).

### 2.9 cDNA library preparation and sequencing

PDCoV-infected Huh7 cells were submitted to Beijing Novogene Co., Ltd. The RNA integrity of the PDCoV-infected Huh7 cells was assessed using the RNA Nano 6000 Assay Kit of the Bioanalyzer 2100 System (Agilent Technologies). mRNA was purified using poly T oligo-attached magnetic beads. The library fragments were purified using the AMPure XP system (Beckman Coulter, United States). The clustering of the index-coded samples was performed on a cBot Cluster Generation System using TruSeq PE Cluster Kit v3-cBot-HS (llumia). After cluster generation, the library preparations were sequenced on an Illumina Novaseq platform and 150 bp paired-end reads were generated.

### 2.10 Read quality control and mapping

All analyses were performed using the clean data. The index of the reference genome was built and paired-end clean reads were aligned to the reference genome using Hisat2 (Version 2.0.5). Feature counts (Version 1.5.0-p3) were performed to determine the number of reads mapped to each gene. Fragments Per Kilobase of exon model per Million mapped fragments (FPKM) were calculated based on the gene length and read count.

### 2.11 Differentially expressed gene analysis

Differentially expressed gene (DEG) analysis was performed using the DESeq2 R package (Version 1.20.0). The resulting *p*-values were adjusted using Benjamini and Hochberg’s approach to control for the false discovery rate. Genes with *p* ≤ 0.05, |log2FoldChange| ≥ 1.0, identified by DESeq2, were assigned as differentially expressed. Gene Ontology (GO) and Kyoto Encyclopedia of Genes and Genomes (KEGG) pathway analyses were performed using the Cluster Profiler R package (Version 3.22.5). Statistical significance was set at *p* < 0.05. The GO and KEGG datasets were used for Gene Set Enrichment Analysis (GSEA). STRING was employed to build a protein–protein interaction (PPI) network for DEGs using an interaction score threshold of 0.40. The score threshold of up-regulated and down-regulated genes are 0.90 and 0.60. PPI networks were visualized using Cytoscape (Version 3.9.1). Reactome, Disease Ontology (DO), and DisGeNET pathways with *p* < 0.05 were considered significantly enriched using Cluster Profiler software.

### 2.12 Quantitative real-time-PCR

Total RNA was extracted from PDCoV-infected Huh7 cells using a TRIzol Kit (Invitrogen) and subjected to reverse transcription using a PrimeScript RT Reagent Kit (Takara). The cDNAs were used as templates to determine the mRNA expression levels using TB Green Premix Ex Taq II (Takara). The human *GAPDH* gene was used for normalization, and the 2^−∆∆CT^ method was used to calculate the relative amounts of the PCR products. The primers used for qRT-PCR are listed in Table 1.

**Table 1:** The primers of selected genes analyzed with qRT-PCR.

Genes	Primer sequences (5′-3′)
PTGS2	Forward primer: GTTCCACCCGCAGTACAGAA Reverse primer: AGGGCTTCAGCATAAAGCGT
CXCL8	Forward primer: GGTGCAGTTTTGCCAAGGAG Reverse primer: TTCCTTGGGGTCCAGACAGA
ATG14	Forward primer: CGCTGTGCAACACTACCCG Reverse primer: TTGCTTGCTCTTAAGTCGGC
MAP3K14	Forward primer: CCCATGCTACAGAGGGCAAA Reverse primer: ATGAGCCAGGGACTTTGAGC
JAK2	Forward primer: TGCCGGTATGACCCTCTACA Reverse primer: ACCAGCACTGTAGCACACTC
HSPA1B	Forward primer: AGCTGGAGCAGGTGTGTAAC Reverse primer: TCCTCAATGGTAGGGCCTGA
MAP2K6	Forward primer: TACGGGGTGGTGGAGAAGAT Reverse primer: CACATCACCCTCCCGAAACA
LRP1	Forward primer: CTGGCGAACAAACACACTGG Reverse primer: CACGGTCCGGTTGTAGTTGA
GAPDH	Forward primer: GGAGCGAGATCCCTCCAAAAT Reverse primer: GGCTGTTGTCATACTTCTCATGG

### 2.13 Statistical analysis

Data were analyzed using an independent-sample *t*-test and expressed as the mean ± standard deviation (SD) of at least three independent samples using GraphPad Prism software (Version 8.0.2). Ns, *p* > 0.05; *, *p* < 0.05; **, *p* < 0.01; ***, *p* < 0.001.

## 3 Results

### 3.1 Virus isolation and characterization

**Figure 1:**
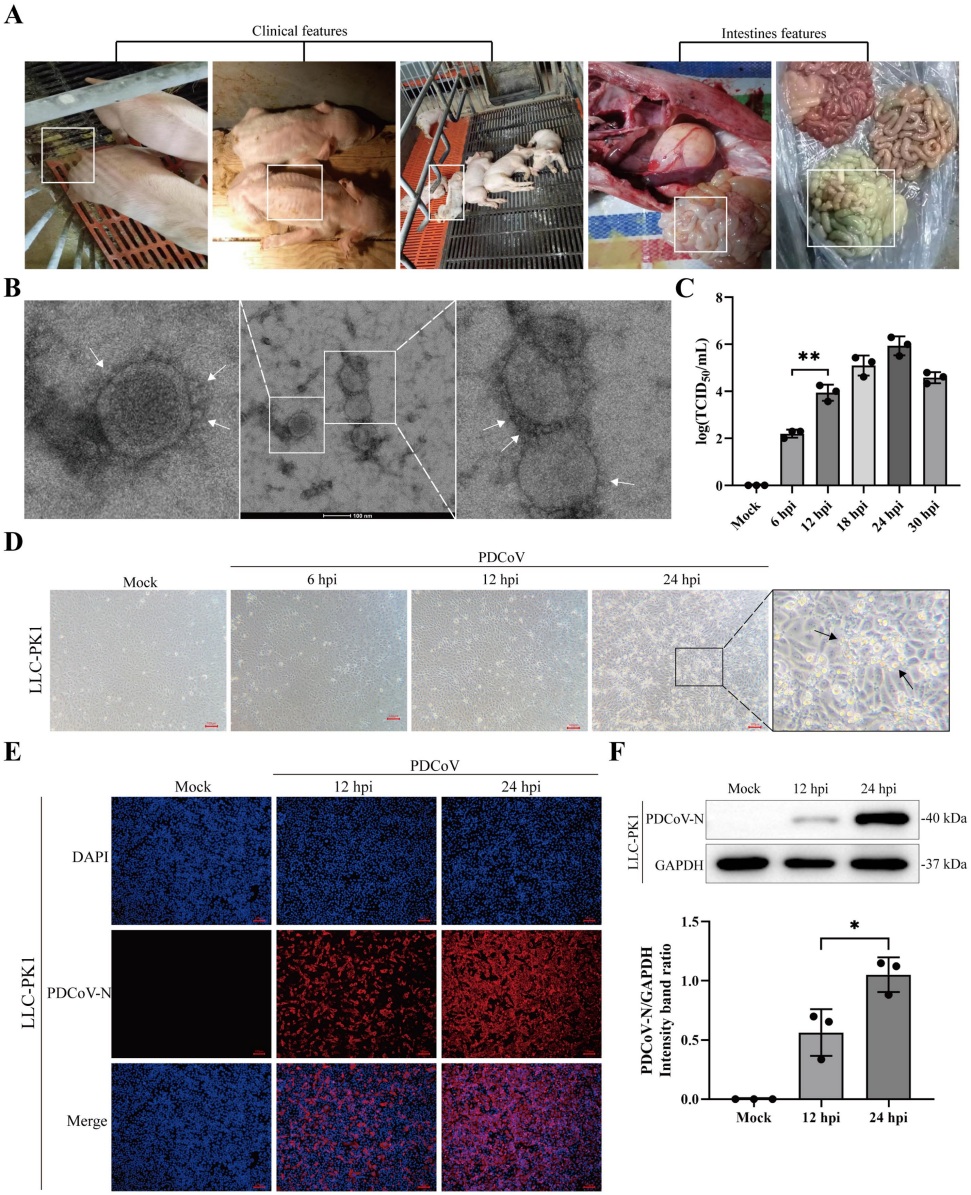
Isolation and characterization of PDCoV CHN/SX-Y/2023. **(A)** Clinical and intestines features of PDCoV-infected piglets were showed in white box. **(B)** Electron microscopic images of purified virus particles in LLC-PK1 cells. Bar, 100 nm. The white arrows represent coronary spike protein outline. **(C)** TCID_50_ titration of LLC-PK1 cells were inoculated with PDCoV (MOI = 1). **(D)** Cytopathic changes of LLC-PK1 cells mock- and infected-PDCoV (MOI = 1) for 6, 12 and 24 h. **(E)** Immunofluorescence of PDCoV was detected in mock- and infected-PDCoV (MOI = 1) for 12 and 24 h. PDCoV N protein was strained red. Nuclei was strained blue with 4’,6-diamidino-2-phenylindole (DAPI). All images were taken at x10 magnification. Bar, 100 nm. **(F)** LLC-PK1 cells was infected with PDCoV (MOI = 1) and the expression levels of PDCoV N and GAPDH at 12 and 24 hpi were detected using western blotting. Statistical significance is determined by *t* test (*p < 0.05, **p < 0.01).

The clinical symptoms of PDCoV-infected piglets included diarrhea, weight loss, and transparent and thin-walled intestines (Figure 1A). The purified virus particles had crown-shaped surface projections with diameters of 100–120 nm (Figure 1B; Supplementary Figure S1A). The proliferation of PDCoV was detected by TCID_50_ and western blotting at different time points (Figure 1C; Supplementary Figures S1B,C). The accumulation of PDCoV N and TCID_50_ appeared with large numbers at 24 hpi (Supplementary Figures 1B,C). The CPE, characterized by rounded, clustered, and increased refraction of LLC-PK1 cells, was evident at 24 h post-infection (hpi) (Figure 1D). Stronger specific red fluorescence was observed in PDCoV-infected cells at 24 hpi (Figure 1E). Meanwhile, western blotting revealed that the accumulation of PDCoV N increased at 24 hpi (Figure 1F). These data demonstrated that the isolated PDCoV strain could propagate in LLC-PK1 cells and the virus titers has similar trends with other PDCoV strain.

### 3.2 Phylogenetic analysis and genomic characterization

**Figure 2:**
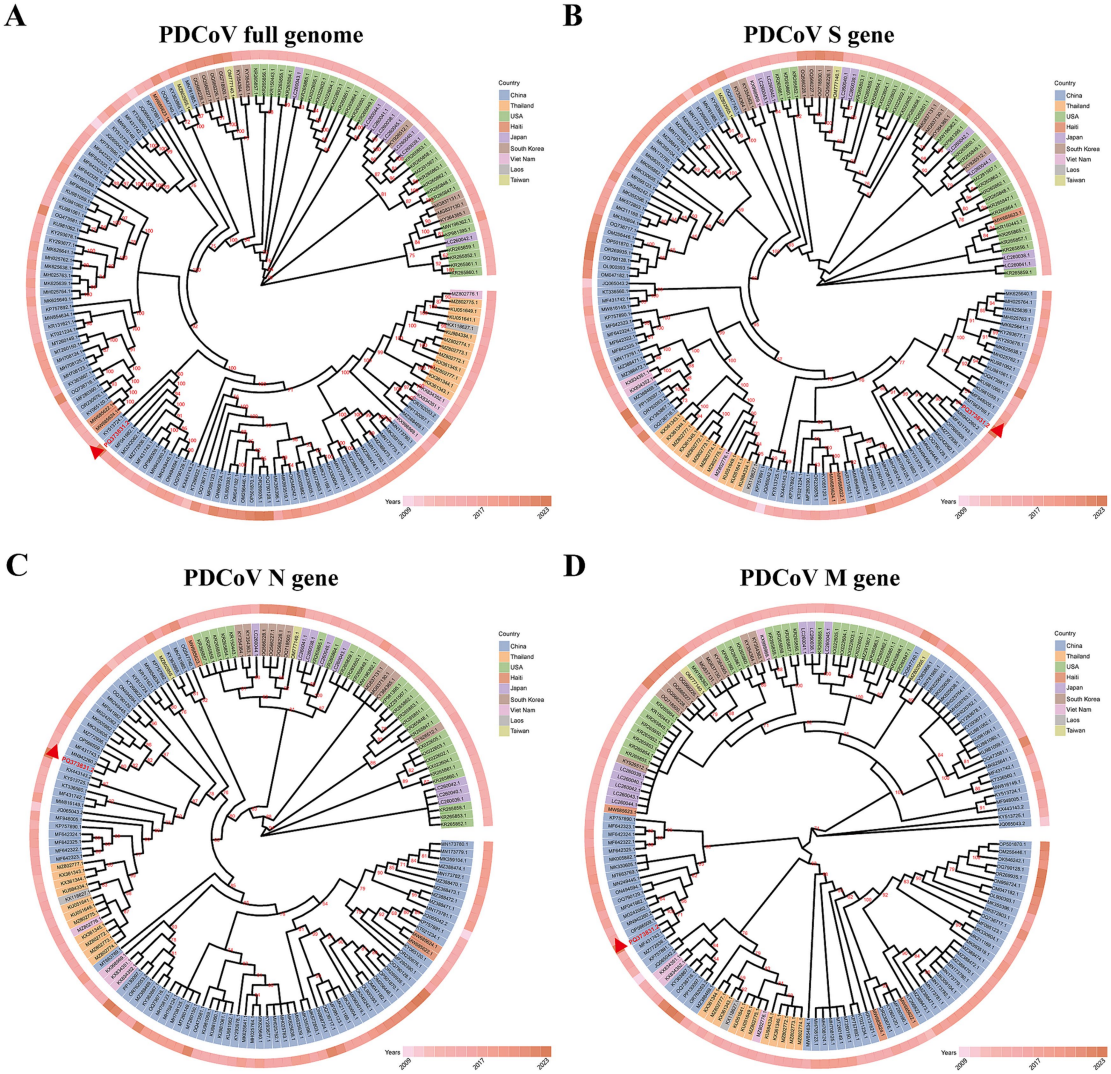
Maximum-likelihood phylogenetic tree based on genome sequences of 154 PDCoV strains. **(A)** Phylogenetic tree based on whole-genome sequences. **(B–D)** Phylogenetic tree based on the S, N, M gene of 154 PDCoV strains, respectively. The different colors identifying the different countries are explained in the legend. Isolated years were indicated by the different degrees of pink. All sequences are identified by GenBank ID. The CHN/SX-Y/2023 strain was marked by red triangle.

Phylogenetic tree analysis of 154 full PDCoV genomes demonstrated that the CHN/SX-Y/2023 strain belongs to the Chinese lineage (Figure 2A). Interestingly, the Haitian strains were closely related to some Chinese strains. PDCoV/Haiti/Human/0256-1/2015 (GenBank ID: MW685623.1) formed an independent cluster, which formed a different branch from the CHN/SX-Y/2023 strain. However, PDCoV/Haiti/Human/0329-4/2015 (GenBank ID: MW685624.1), together with PDCoV/Haiti/Human/0081-4/2014 (GenBank ID: MW685622.1), showed 100% similarity to CHN/Tianjin/2016 (GenBank ID: KY065120.1), which shares a common ancestor with the new PDCoV strain. The phylogenetic tree based on the S gene revealed that the CHN/SX-Y/2023 and HeN/Swine/2015 (GenBank ID: MN942260.2) strains belong to the same branch (Figure 2B), whereas it clustered with the HeN/Swine/2015 and SD (GenBank ID: MF431743.1) strains in the N-based tree (Figure 2C). The results of M gene analysis indicated that the CHN/SX-Y/2023 strain clustered with the SD and BN (GenBank: MZ772936.1) strains (Figure 2D). Notably, phylogenetic trees constructed using the entire genome and the S, N, and M gene sequences showed different clustering patterns, suggesting that the genetic diversity in PDCoV is geographically and temporally distributed.

**Figure 3:**
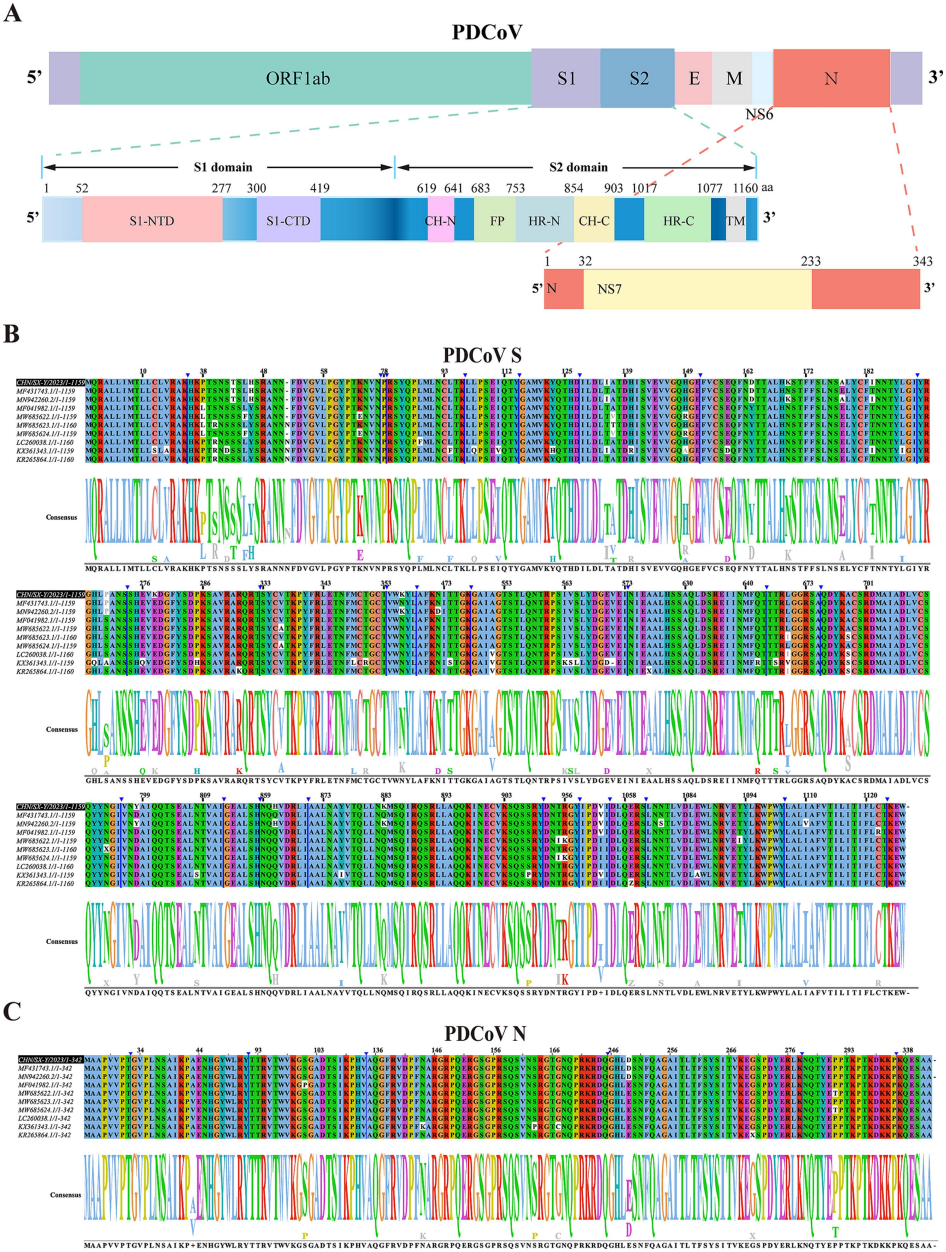
Genomic characterization and comparative analysis amino acid sequence of PDCoV CHN/SX-Y/2023. **(A)** Visualization of PDCoV complete genome sequence. The spike protein contains S1 and S2 subunit. S1-NTD and S1-CTD are N-terminal and C-terminal domain of S1. CH-N and CH-C, central helices N and C. HR-N and HR-C, heptad repeats N and C. FP, fusion peptide. **(B,C)** The multiple sequence alignment based on the S and N gene of PDCoV strains. Partially identical nucleotides are combined and the white boxes represent different nucleotides. The black box represents the CHN/SX-Y/2023 strain.

The CHN/SX-Y/2023 strain contained 25,415 nucleotides (nt), characterized by the following gene order: 5′-UTR-ORF1ab-S-E-M-NS6-N-3′UTR. The S protein ectodomain consisted of S1 and S2 subunits, with the receptor-binding domain (RBD, S1-CTD) located in the S1 subunit (Figure 3A). Nonstructural gene 6 (NS6) was located between M and N, and nonstructural gene 7 (NS7) was located within the N gene (Figure 3A). The coding potential and putative transcriptional regulatory sequences of CHN/SX-Y/2023 are listed in Supplementary Table S4. To better understand the evolutionary characteristics, we further investigated the sequence similarities of the S, RBD, N, and M genes from China (GenBank: MF431743.1, MF041982.1, and MN942260.2), Haiti (GenBank: MW685622.1, MW685623.1, and MW685624.1), United States/Minnesota (GenBank ID: KR265864.1), Japan (GenBank ID: LC260038.1), and Thailand (GenBank ID: KX361343.1) strains. The S amino acid sequences of the CHN/SX-Y/2023 PDCoV strain were 98.7–99.2% identical to those of the Chinese PDCoV strains, 98.0–98.3, 98.3, and 98.4% identical to the Haitian, Minnesota, United States, and Japanese PDCoV strains, respectively, and shared the lowest homology (96.4%) with the Thai strains (Figure 3B; Supplementary Table S5). Moreover, the RBD amino acid sequence of CHN/SX-Y/2023 was 100% identical to that of HeN/Swine/2015, and only had a mutation site (N396K) different from other strains, including the Chinese, Haitian (GenBank ID: MW685623.1), Minnesota, United States, and Japanese strains (Supplementary Figure S2A; Supplementary Table S5). In addition, the CHN/SX-Y/2023 PDCoV strain had mutation sites (A335V and N396K) in common with Haitian strains (GenBank ID: MW685622.1 and MW685624.1), and L347M and R349T with Thai strains (Supplementary Figure 2A). The N and M genes shared 98.8–100% and 99.5–100% identities with other PDCoV strains, respectively (Figure 3C; Supplementary Figure S2B). These results of N gene showed that the CHN/SX-Y/2023 PDCoV strain was the same as the Haitian isolate MW685623, except for E249D (Figure 3C). However, it was also similar to that of the Haitian isolates MW685622 and MW685624, except for T291P (Figure 3C). The M amino acid sequences contained only three mutation sites, T49A, A57X, and I80T, in MN942260, KR265864, and KX361343, respectively (Supplementary Figure S2B). The PDCoV N and M proteins are highly conserved compared with the S protein.

### 3.3 Cell type susceptibility

**Figure 4:**
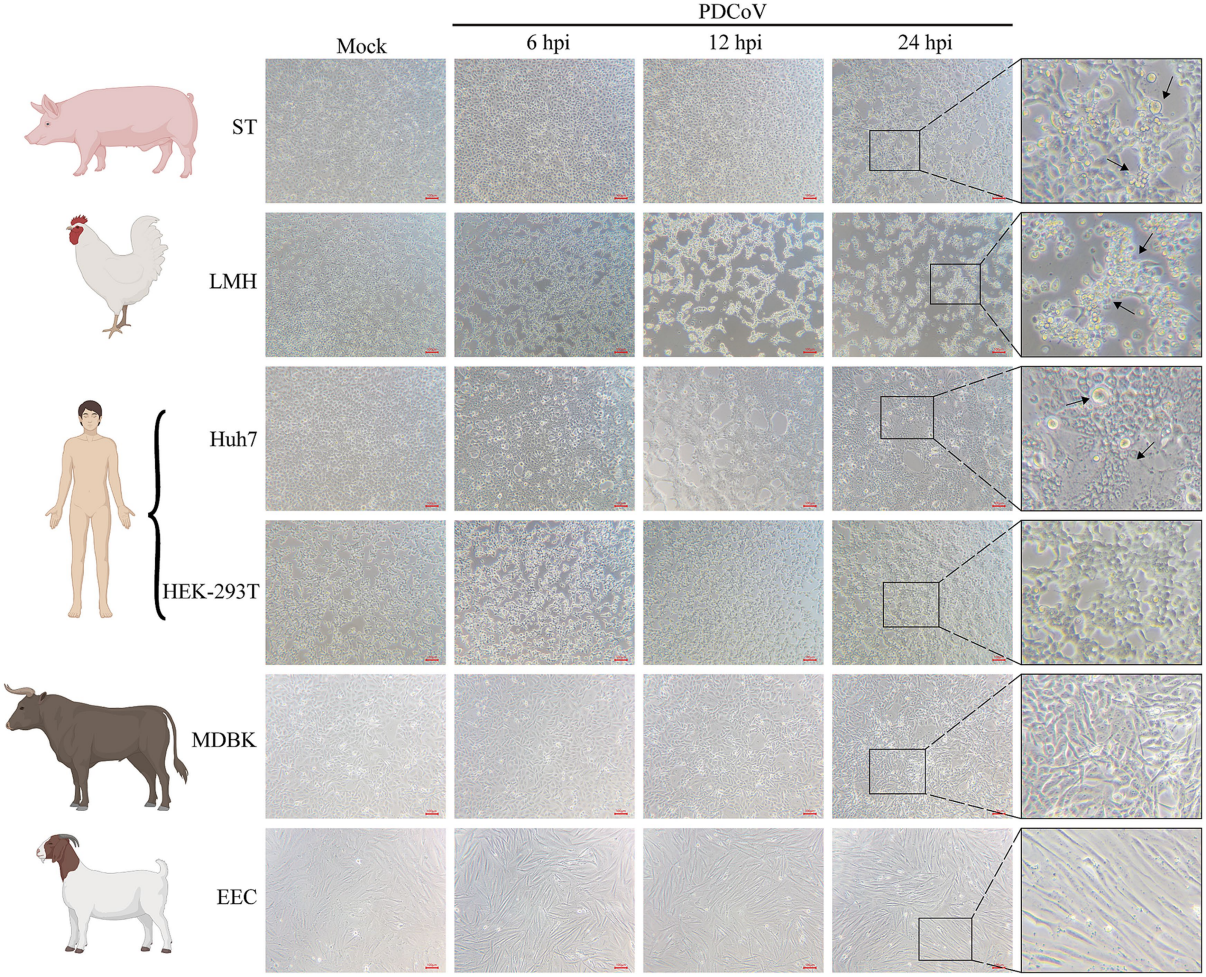
Cytopathic changes of ST, Huh7, LMH, HEK-293T, MDBK, and EEC infected with mock- and infected-PDCoV (MOI = 1) for 6, 12 and 24 h. The black arrows represent obvious cytopathic changes. Bar, 100 nm. All images were taken at x10 magnification.

Cells from different species were used to determine their susceptibility to PDCoV infection at 6, 12, and 24 h. CPE, characterized by rounded, clustered, and increased refraction of ST and LMH cells, was evident at 24 hpi (Figure 4). PDCoV caused significant cell lysis, shedding, enlargement, and membrane fusion in Huh7 cells at 24 hpi (Figure 4). The apoptotic rates of these cells increased in a time-dependent manner (Figure 4). However, no obvious CPE was observed in HEK-293T, MDBK, or EEC cells at 24 hpi (Figure 4). Moreover, no detectable changes were observed in PDCoV-infected BHK-21, MDCK, or Vero-CCL81 cells at 24 hpi (Supplementary Figure S3A).

**Figure 5:**
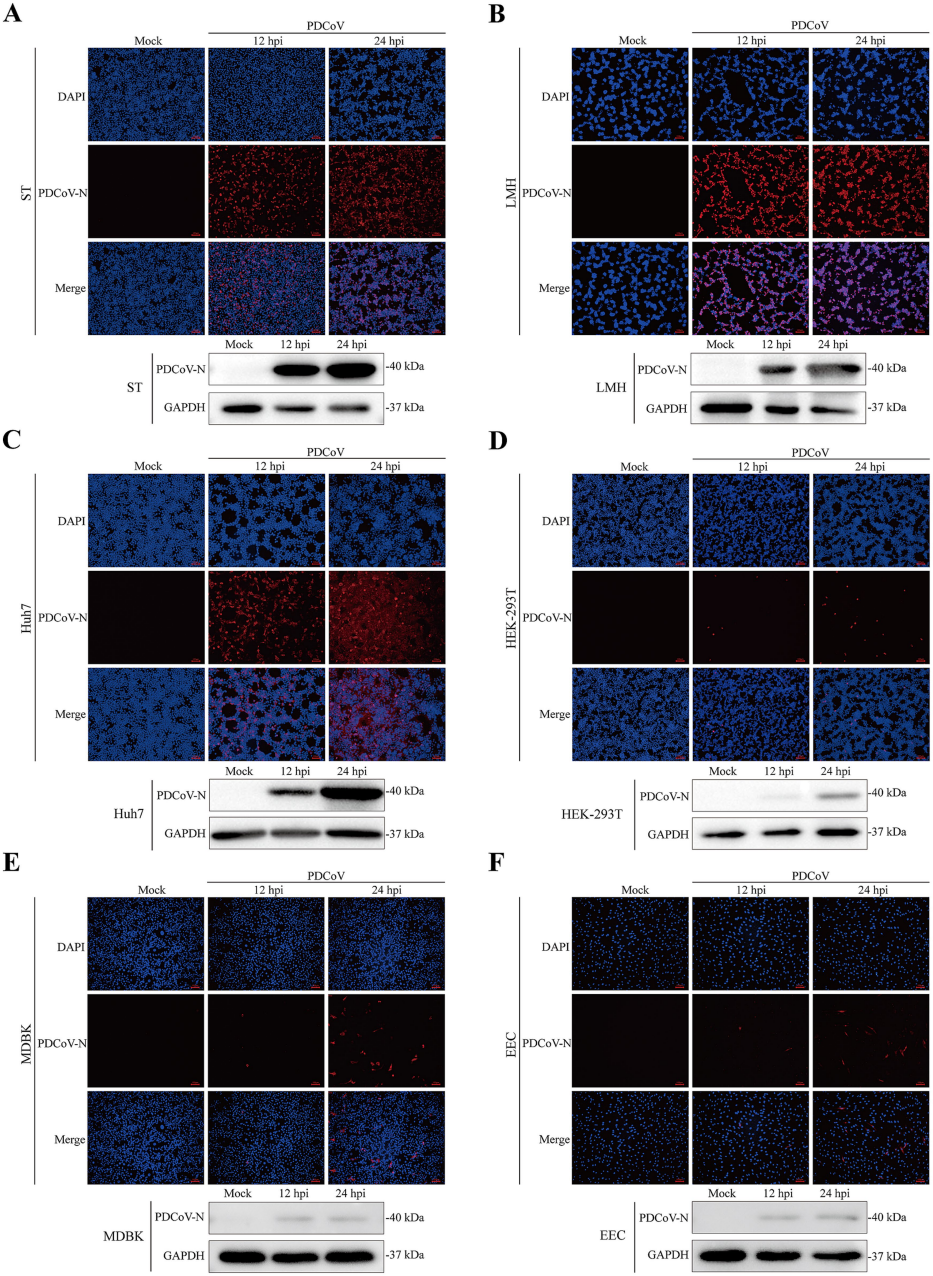
Immunofluorescence and western blotting assay of PDCoV were detected in mock- and infected-PDCoV different cell lines (MOI = 1) at 12 and 24 hpi. **(A–F)** ST, LMH, Huh7, HEK-293T, MDBK, EEC cells. Bar, 100 nm. The PDCoV N was detected to prove the infection of the virus. GAPDH was as the internal reference. Nuclei was strained blue with DAPI. All images were taken at x10 magnification.

The immunofluorescence assay revealed specific red fluorescence indicating that the PDCoV N protein was clearly observed in ST, LMH, and Huh7 cells at 12 and 24 hpi (Figures 5A–C). Minimal red fluorescence was observed in HEK-293T, MDBK, EEC, and Vero-CCL81 cells following PDCoV infection (Figures 5D–F; Supplementary Figure S3C). However, red fluorescence was not observed in BHK-21 or MDCK cells (Supplementary Figures S3B,D).

Western blotting indicated that the expression of the PDCoV N protein was significantly increased in ST, LMH, and Huh7 cells at 24 hpi (Figures 5A–C). PDCoV N protein was detected in HEK-293T, MDBK and EEC cells, with no obvious changes at 12 and 24 hpi (Figures 5D–F). Notably, PDCoV N protein expression was not detected in MDCK cells (Supplementary Figure S3D).

In conclusion, the PDCoV CHN/SX-Y/2023 strain can infect and proliferate in a wide range of cell lines, including ST, Huh7, and LMH cells. However, HEK-293T, EEC, MDBK, and Vero-CCL81 cells were not found to be susceptible. BHK-21 and MDCK cells could not be infected in this study.

### 3.4 Differentially expressed genes analysis

**Figure 6:**
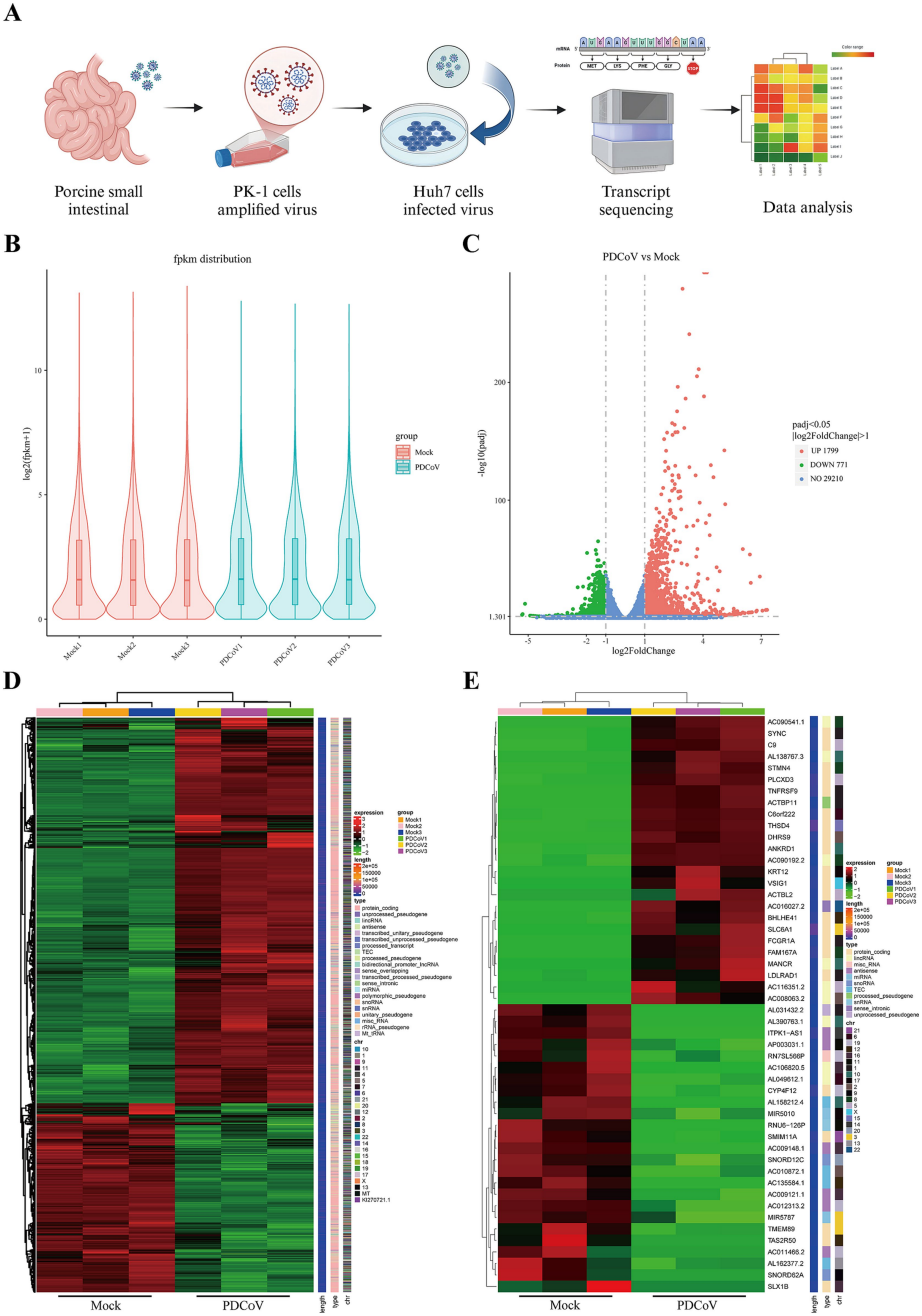
The genes were differentially expressed PDCoV CHN/SX-Y/2023 infected Huh7 cells in 24 h. **(A)** Workflow and screening strategy of DEGs analysis. **(B)** The violin plot of gene expression patterns for each sample with the origin representing the median. **(C)** The volcano map of DEGs, blue represents non-differentially expressed genes, red represents up-regulated DEGs and green represents down-regulated DEGs. **(D)** Clustering heat map of DEGs. Expression levels in the heatmaps are color coded from green (low) to red (high). **(E)** Clustering heat map of top 50 DEGs, including 25 upregulated and downregulated genes, respectively.

The screening strategy for the transcriptome libraries constructed from mock- and PDCoV-infected Huh7 cells are shown in Figure 6A. In this study, approximately 44.41 million clean reads remained after screening, and the percentages of clean data for Q20 and Q30 were >98 and 95%, respectively. Moreover, the GC content of the clean reads in each sample ranged from 46.76 to 48.99% (Supplementary Table S6). These clean reads were of high quality and suitable for analysis. Simultaneously, based on the distribution of gene expression after quantification of gene expression levels as FPKM, the samples in each group were repeatable and similar samples clustered together (Figure 6B). Notably, 2,570 DEGs were identified in PDCoV-infected Huh7 cells, of which 1799 were upregulated and 771 were downregulated (Figure 6C). Cluster analysis revealed distinct trends in the expression of genomic transcripts in PDCoV-infected Huh7 cells at 24 hpi compared to that in mock-Huh7 cells (Figure 6D). The top 25 DEGs identified through clustering and heatmap analyses were associated with inflammatory cytokines, lipid catabolic processes, and immunity, including *TNFRSF9*, *PLCXD3*, *DHRS9*, *SMM11A*, *FCGR1A*, and *VSIG1* (Figure 6E). Notably, the ferroptosis-associated marker *PTGS2* was upregulated in the top 50 DEGs (Supplementary Table S7). These key genes may be closely associated with PDCoV infection.

### 3.5 Enrichment analysis of the DEGs

**Figure 7:**
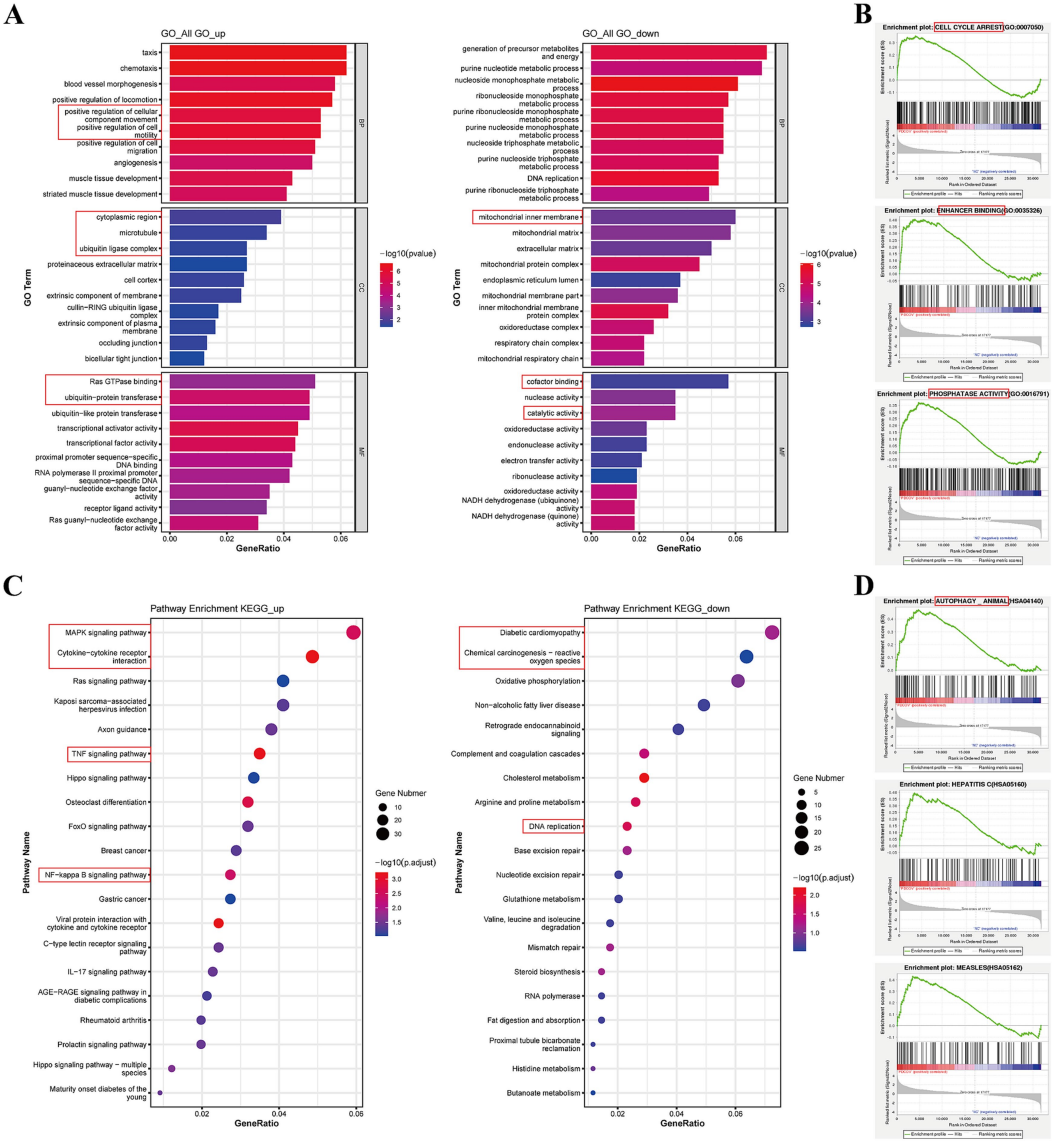
GO and KEGG analysis for the DEGs between PDCoV-infected and mock-infected Huh7 cells. **(A)** Top 10 of enriched GO terms for up-regulated and down-regulated DEGs. The x-axis represents the GeneRatio and the y-axis was the GO function. **(B)** GSEA of enriched GO. The abscissa represents the rank in ordered dataset and the ordinate represents enrichment score (ES). **(C)** Top 20 of enriched KEGG pathway for up-regulated and down-regulated DEGs. The x-axis was GeneRatio and the y-axis was the pathway. **(D)** GSEA of enriched KEGG. Circles indicate numbers of enriched genes and colors depict the *p*-value coded from blue (low) to red (high).

GO analysis categorized genes into biological processes (BPs), cellular components (CCs), and molecular functions (MFs). For BPs, the upregulated DEGs were mainly enriched in cellular component movement and cell motility, whereas downregulated DEGs were mainly enriched in metabolic processes (Figure 7A). For CCs, the upregulated DEGs were mainly enriched in the cytoplasmic region, microtubules, and ubiquitin ligase complex, whereas downregulated DEGs were related to the mitochondrial inner membrane (Figure 7A). For MFs, the upregulated DEGs were related to Ras GTPase binding and ubiquitin protein transferase, whereas downregulated DEGs were related to cofactor binding and catalytic activity (Figure 7A). For all GO analyses, positive regulation of cell motility in BPs, proteinaceous extracellular matrix in CCs, and proximal promoter sequence-specific DNA binding, transcription factor activity, and receptor regulator activity in MFs were enriched (Supplementary Table S8). GSEA data indicated that cell cycle arrest, enhancer binding, and phosphatase activity were enriched (Figure 7B; Supplementary Table S9).

KEGG analysis indicated that DEGs were mostly involved in canonical pathways, such as the MAPK, JAK–STAT, TNF, and NF-κB signaling pathways, cellular senescence, and viral protein interactions with cytokines and cytokine receptors (Table 2). The upregulated DEGs were enriched in the MAPK signaling pathway, cytokine-cytokine receptor interaction, TNF, and NF-κB signaling pathways (Figure 7C). However, downregulated DEGs were mainly enriched in diabetic cardiomyopathy, chemical carcinogenesis-reactive oxygen species, and DNA replication (Figure 7C). GSEA of the KEGG results revealed the AMPK, TGF beta, phospholipase D, autophagy, and endocytosis signaling pathways (Supplementary Table S10; Figure 7D). These data suggest that PDCoV infection can induce a response from the host immune system to resist viral invasion.

**Table 2:** Top 20 of KEGG pathways analysis in PDCoV infected Huh7 cells at 24 hpi.

KEGG ID	Description	Gene ratio	*p* value	Gene name
hsa04010	MAPK signaling pathway	49/1003	0.000582	DUSP1/AREG/JUN/FGF19/TGFBR2 et al.
hsa04060	Cytokine-cytokine receptor interaction	39/1003	5.06E-05	CXCL5/BMP2/CXCL1/IL6R/TNFRSF11B et al.
hsa05167	Kaposi sarcoma-associated herpesvirus infection	35/1003	0.008068	ATG14/CXCL1/JUN/MAP2K6/JAK1 et al.
hsa04360	Axon guidance	29/1003	0.018164	ABLIM3/SEMA3C/SEMA6A/NTN4/DPYSL2 et al.
hsa04218	Cellular senescence	28/1003	0.022746	CDKN2B/SQSTM1/CXCL8/TGFBR2/CAPN2 et al.
hsa05202	Transcriptional misregulation in cancer	28/1003	0.031956	CDKN2B/SQSTM1/CXCL8/TGFBR2/MAP2K6 et al.
hsa04668	TNF signaling pathway	27/1003	0.000101	CXCL5/CREB5/CXCL1/TNFAIP3/BIRC3 et al.
hsa04390	Hippo signaling pathway	27/1003	0.031811	AREG/BMP2/FZD5/BIRC3/PRSS23 et al.
hsa05224	Breast cancer	26/1003	0.001498	FZD5/PRSS23/JUN/FRAT2/FGF19 et al.
hsa05226	Gastric cancer	26/1003	0.002003	FZD5/CDKN2B/PRSS23/FRAT2/FGF19 et al.
hsa04380	Osteoclast differentiation	24/1003	0.000574	JUN/SQSTM1/TGFBR2/MAP2K6/JAK1 et al.
hsa04068	FoxO signaling pathway	24/1003	0.021135	CDKN2B/PLK2/TGFBR2/SIRT1/CDK2 et al.
hsa04630	JAK–STAT signaling pathway	22/1003	0.024464	MCL1/IL6R/JAK1/PIK3CB/IL15 et al.
hsa04936	Alcoholic liver disease	21/1003	0.029143	CXCL1/LPIN2/CXCL8/CXCL2/MAP2K6 et al.
hsa04550	Signaling pathways regulating pluripotency of stem cells	21/1003	0.04824	FZD5/PRSS23/FZD7/JAK1/FGFR4 et al.
hsa04064	NF-kappa B signaling pathway	20/1003	0.001801	CXCL1/TNFAIP3/BIRC3/CXCL8/CXCL2 et al.
hsa04726	Serotonergic synapse	19/1003	0.017172	DUSP1/MAOB/HTR1B/GNG2/SLC6A4 et al.
hsa04625	C-type lectin receptor signaling pathway	18/1003	0.01747	JUN/PIK3CB/RELB/PLCG2/MAP3K14 et al.
hsa04061	Viral protein interaction with cytokine and cytokine receptor	16/1003	0.000607	CXCL5/CXCL1/IL6R/CXCL8/CXCL2 et al.
hsa04610	Complement and coagulation cascades	16/1003	0.007169	FGA/SERPINF2/FGB/SERPINA1/F7 et al.

**Figure 8:**
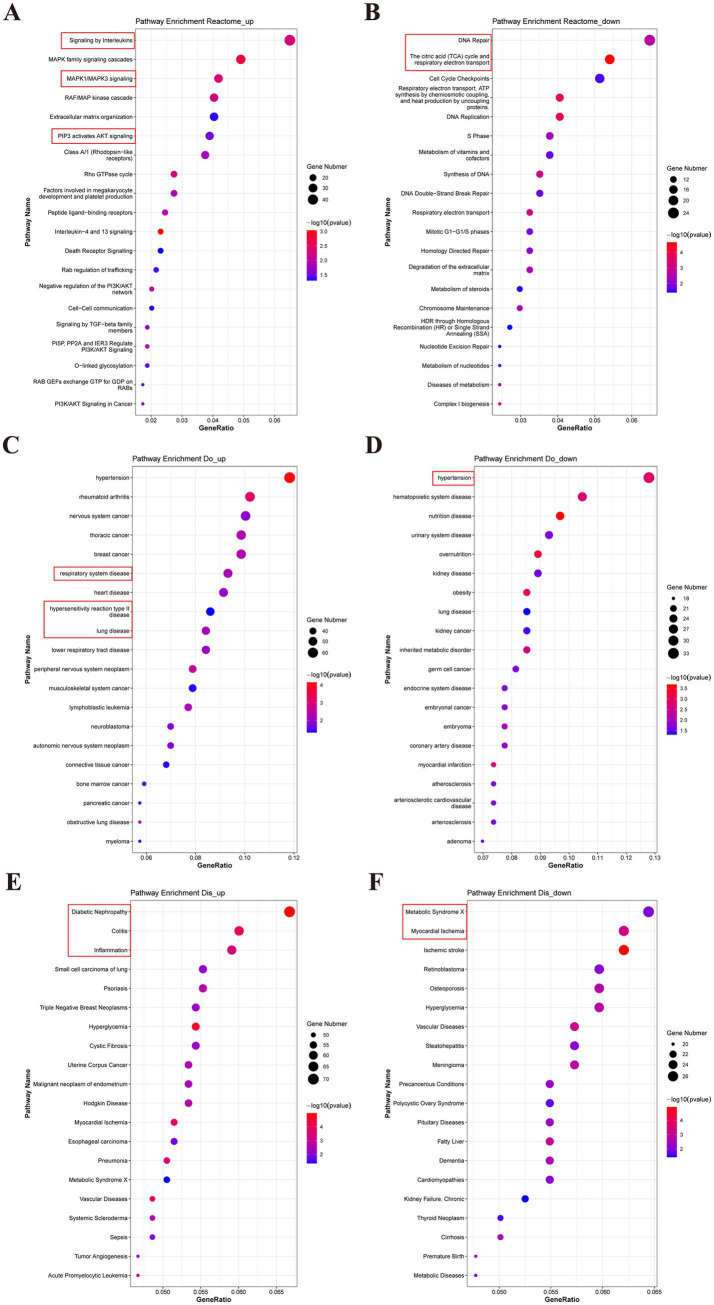
Reactome, Do and DisGeNET analysis for the DEGs between PDCoV-infected and mock-infected Huh7 cells. **(A,C,E)** Top 20 of enriched Reactome, DO, DisGeNET terms for up-regulated DEGs. **(B,D,F)** Top 20 of enriched Reactome, DO, DisGeNET terms for down-regulated DEGs. The abscissa represents the enrichment GeneRatio and the ordinate represents the pathway. Expression levels in the maps are color coded from blue (low) to red (high). Circles indicate numbers of enriched genes.

To gain a comprehensive understanding of the various reactions, biological pathways, and disease-related genes in the human model species, the Reactome, DO, and DisGeNET databases were used. Our analysis revealed that the upregulated DEGs were related to the inflammatory response and cellular programs, including signaling by interleukins (IL-1 to IL-38), MAPK signaling, and PIP3 activation of AKT signaling. Conversely, the downregulated DEGs were mainly involved in DNA repair, the citric acid cycle, and respiratory electron transport in the Reactome data (Figures 8A,B). Furthermore, DO analysis indicated that DEGs related to the respiratory system, hypersensitivity reaction type II, and lung disease were upregulated, whereas those related to hypertension and hematopoietic system disease were downregulated (Figures 8C,D). DisGeNET analysis showed that upregulated DEGs were enriched in diabetic nephropathy, colitis, and inflammation, while downregulated DEGs were related to metabolic syndrome X and myocardial ischemia (Figures 8E,F). The top 15 results from the Reactome, DO, and DisGeNET databases implied that the DEGs were related to RAF-independent MAPK1/3 activation, interleukin-10 signaling, inflammation, and lower respiratory system disease (Supplementary Table S11). The data from the Reactome database were consistent with the KEGG analysis. These findings provide novel insights into the disease-related reactions and pathways associated with PDCoV infection in humans.

### 3.6 PPI network analysis

**Figure 9:**
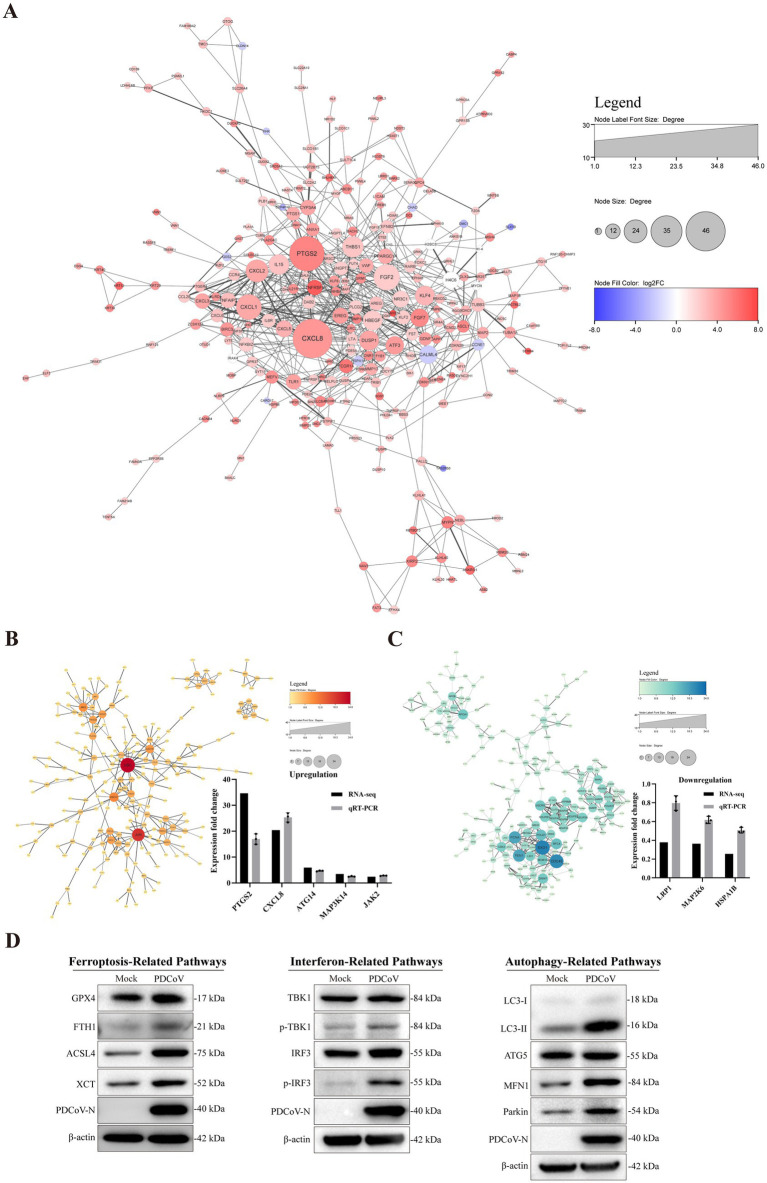
Interacting network of differentially expressed proteins. **(A)** PPI network of the DEGs. **(B,C)** PPI network of up-regulated and down-regulated DEGs and RT-qPCR verified results. **(D)** The related protein expression level of ferroptosis, autophagy and immune response in the context of PDCoV infection using western blotting. β-actin was used as an internal reference. All PPI networks were based on STRING analysis. Each node represented a protein, and each edge represented the interaction between proteins. The upregulated proteins are shown in red shadow, and the downregulated proteins are shown in blue.

The network interaction diagram highlights *CXCL8*, *IL15*, *PTGS2*, *DUSP1*, *ATF3*, and *PPARGC1*, which are associated with inflammation, immune responses, cellular stress responses, and lipid metabolism (Figure 9A). The upregulated DEGs, including *UBC*, *JUN*, *SQSTM1*, *JAK2*, *PIK3CB*, *PLCG2*, *CXCL1*, and *PPARGC1A*, were associated with inflammation, autophagy, immune responses, and lipid metabolism (Figure 9B). In contrast, the significantly downregulated genes were *EXO1*, *CDC45*, *MCM10*, *FEN1*, *NDUFB1*, and *POLR2F*, which are related to cell division, DNA reproduction, electron transfer in mitochondria, and messenger RNA synthesis in eukaryotes (Figure 9C). Moreover, *PTGS2*, *CXCL8*, *ATG14*, *MAP3K14*, *JAK2*, *HSPA1B*, *MAP2K6*, and *LRP1* were consistent with the sequencing results, showing the same relative regulation patterns of DEGs between the two methods. Numerous genes related to autophagy and immune responses were significantly altered in response to PDCoV infection.

Based on the data presented above, we analyzed the expression levels of proteins related to ferroptosis, autophagy, and immune responses in the context of PDCoV infection. Western blotting results indicated that PDCoV infection increased the expression of proteins related to ferroptosis, such as GPX4, FIH1, ACSL4, and XCT (Figure 9D). Additionally, the levels of MFN1, Pakin, and LC3-II, which are related to autophagy, increased following PDCoV infection, but there were no detectable changes in ATG5 levels (Figure 9D). The expression levels of p-TBK1 and p-IRF3, which are related to the interferon pathway, were increased (Figure 9D). These data indicate that PDCoV infection induces changes in proteins related to ferroptosis, autophagy, and immune responses in host cells.

## 4 Discussion

CoVs have repeatedly crossed the host barrier between various animals, such as swine acute diarrhea syndrome coronavirus (SADS-CoV) from bats to swine (Hassanin et al., 2024), and severe acute respiratory syndrome coronavirus 2 (SARS-CoV-2) from animal reservoirs to humans (Rakhmetullina et al., 2024). PDCoV, PEDV, TGEV, and PoRV epidemics are commonly accompanied by co-infections and secondary infections, which contribute to increased morbidity and mortality in herds (Yan et al., 2022; Zhu et al., 2021). Three blood samples were found to be infected with PDCoV in Haitian children (Lednicky et al., 2021), suggesting a risk to public health. However, there are currently no effective treatments or commercially available vaccines for the prevention and control of PDCoV infection (Miao et al., 2023; Zhu M. et al., 2023; Zhu X. et al., 2023).

In this study, the PDCoV CHN/SX-Y/2023 strain caused diarrhea and severe enteritis in piglets, similar to other PDCoV strains. The CHN/SX-Y/2023 strain exhibited the typical genome organization and structural characteristics of coronaviruses. Phylogenies showed that, based on the S and N gene trees, our isolated PDCoV was classified into the Chinese lineage, which belongs to the same branch as the HeN/Swine/2015 strain. Furthermore, the amino acid sequence of the RBD of CHN/SX-Y/2023 was identical to that of HeN/Swine/2015. A previous study indicated that globally, PDCoVs consist of the United States/Japan/South Korea, China, Vietnam, Laos, and Thailand lineages (Yang et al., 2017). Both the Chinese and American lineages are major genotypes worldwide (He et al., 2020; Guo et al., 2024). The CHN/SX-Y/2023 strain was highly similar to the HeN/Swine/2015 strain from Henan Province, China. Geographical proximity may have facilitated PDCoV transmission between Henan and Shanxi Provinces. It is worth noting that Haitian human-infecting strains (GenBank ID: MW685622.1 and MW685624.1) belonged to the same branch as the CHN/SX-Y/2023 strain. This raises a fundamentally interesting question regarding whether PDCoV spread and infection increases the risk of viral transmission among humans.

A key step in cross-species transmission is the ability of the virus to interact with host receptors via the spike protein in CoVs (Shang et al., 2018; Li et al., 2019). The S gene of coronaviruses is associated with tissue tropism and host specificity (Liu et al., 2021). In the present study, the amino acid residues Trp-395 (W), Lys-396 (K), and Tyr-397 (Y) were located in the RBD of the CHN/SX-Y/2023 strain. Previous studies have shown that K396 mutations may alter receptor specificity, and consequently, tissue tropism (Zhu et al., 2018; Ji et al., 2022), and that Trp-396 (W) and Tyr-398 (Y) are important for the binding of PDCoV S1 to pAPN (Liu et al., 2021). These sites represent key residues for PDCoV replication that may enhance dynamic movement and accelerate viral membrane fusion events and transmission.

Our findings indicated that the PDCoV CHN/SX-Y/2023 strain can infect cell lines of different species. LLC-PK1, ST, Huh7, and LMH cells were susceptible to our PDCoV strain. HEK-293 T, EEC, MDBK, and Vero-CCL81 cells were non-susceptible, while BHK-21 and MDCK cells were not infected. Previous studies have shown that PDCoV infects piglets, calves, chickens, and mice and exhibits a broad host range (Xia et al., 2023; Jung et al., 2017; Liang et al., 2019). PK15, LLC-PK1, and ST cells are suitable for the steady multiplication of PDCoV, but HEK-293T, BHK-21, and Vero cells are non-susceptible (Jiang et al., 2024; Ma et al., 2024). LMH, DF-1, and Huh7 cells appear to be susceptible to PDCoV infection (Li et al., 2018). These results suggest that the infection characteristics of the CHN/SX-Y/2023 strain are comparable to those of other PDCoV strains; however, different strains may exhibit variations in their interactions with host cells. In addition, PDCoV strains have been isolated from blood samples of Haitian children in 2021 (Lednicky et al., 2021). Recent reports have indicated that human intestinal epithelial cells exhibit a more pronounced response to PDCoV infection than porcine intestinal epithelial cells (Cruz-Pulido et al., 2021). Huh7 and HeLa cells are susceptible to PDCoV, while human lung carcinoma cells (A549) support PDCoV replication in the presence of trypsin (Fang et al., 2021). These observations illustrate that PDCoV not only causes significant damage to pigs, but also poses a potential threat to mammals because of its zoonotic characteristics. In our study, PDCoV causes obviously cytopathic effects in Huh7 cells. So Huh7 cells was selected as models in transcriptome analysis. However, only viral infection was detected following the initial inoculation, and verification is needed to determine whether the virus can be stably passaged in EEC and MDBK.

In recent years, transcriptomics has been widely employed to evaluate host cell responses to viral infections (Gao et al., 2020; Li et al., 2023; Zhu M. et al., 2023; Zhu X. et al., 2023). The role of HSP90AB1 was investigated using comparative transcriptome analysis of PDCoV infection (Zhao et al., 2022). Integrated metabolomic and transcriptomic analyses have revealed that deoxycholic acid promotes TGEV infection by inhibiting the phosphorylation of NF-κB and STAT3 (Zhou et al., 2024). The transcriptional landscape of LLC-PK1 cells infected with PDCoV showed that DEGs were enriched in MAPK pathway (Liu et al., 2024). PDCoV infection in IPEC-J2 cells regulates gene sets associated with cytokine-cytokine receptor interactions and MAPK signaling pathways (Wang et al., 2024). The research findings elucidated that the innate immune-associated genes and signaling pathways in PK-15 cells could be modified by the infection of PDCoV (Jiang et al., 2019). PDCoV infection activates NF-κB signaling pathway and leads to the expression of inflammatory factors in ST cells (Jin et al., 2021). Reported studies indicated that the association between PDCoV infection and innate immunity. TGEV induces inflammatory responses via the RIG-I/NF-κB/HIF-1α/glycolysis axis in intestinal organoids and *in vivo* (Zhang et al., 2024). PDCoV infection remarkably inhibits Sendai virus-induced IFN-λ1 production by suppressing the transcription factors IRF and NF-κB in porcine intestinal mucosal epithelial cells (Liu et al., 2020). In this study, *TNFRSF9, PTGS2, FCGR1A, PLCXD3*, and *DHRS9*, which are associated with inflammatory cytokines, immunity, and lipid catabolic process signals, were identified in PDCoV-infected Huh7 cells. The significantly enriched pathways were related to immune and inflammatory response-associated pathways, such as the MAPK, JAK–STAT, and NF-κB signaling pathways. Moreover, It is imperative to understand the molecular mechanisms underlying PDCoV-induced immune and inflammatory responses. Our findings shed light on the molecular underpinnings of PDCoV infection in Huh7 cells.

Notably, general difference analysis (GO and KEGG) often focuses on comparing gene expression differences between two groups, which may easily miss some genes that are not significantly differentially expressed but have important biological significance. While GSEA does not need to specify a clear differential gene threshold, its algorithm was based on the overall trend of the actual data. In our study, the results of the GSEA of the KEGG pathway indicated significant enrichment in autophagy and endocytosis. Additionally, PPI results indicated that *SQSTM1*, which is related to autophagy, was upregulated. Meanwhile, the expression levels of autophagy-related proteins such as MFN1, Pakin, and LC3-II increased following PDCoV infection. These findings are consistent with those of previous studies, indicating that PDCoV infection induces autophagy (Li et al., 2024; Chen and Burrough, 2022). We also found that PDCoV infection increased the expression of ferroptosis-related proteins such as FIH1, ACSL4, and XCT. Exogenous addition of the ferroptosis activator erastin significantly inhibits PDCoV replication (Wang et al., 2024). Ergosterol peroxide suppresses PDCoV-induced autophagy by inhibiting PDCoV replication via the p38 signaling pathway (Duan et al., 2021). These data indicate that PDCoV infection induces changes in the ferroptosis signaling pathway in host cells. Overall, these findings support the credibility of our transcriptome analysis, and highlight promising avenues for future antiviral research. However, the mechanism by which different PDCoV strains manipulates the autophagy- or ferroptosis-related pathway need to be confirmed on different cells.

In this study, a PDCoV (CHN/SX-Y/2023) strain was successfully isolated, identified, and used to infect different cell lines, and transcriptome analysis was performed in Huh7 cells. The upregulated genes *FCGR1A*, *TNFRSF9*, and *PLCXD3* are associated with immunity, inflammation, and lipid catabolic processes. Notably, PDCoV infection regulated MAPK, TNF, and NF-κB signaling pathways, and viral protein interaction with cytokines and cytokine receptors, and may cause changes in autophagy-related and ferroptosis-related pathways. Our research provides novel insights into the diversification, evolution, characteristics, and interspecies transmission of PDCoV.

## Data Availability

The datasets presented in this study can be found in online repositories. The names of the repository/repositories and accession number(s) can be found below: the National Center for Biotechnology Information, Gene Expression Omnibus (GEO) Accession Number: GSE286021.
