# Correction: Isolation, phylogenetics, and characterization of a new PDCoV strain that affects cellular gene expression in human cells

**DOI:** 10.3389/fmicb.2025.1646820

**Published:** 2025-09-08

**Authors:** Xiaozhu Yang, Hanwei Yin, Mengyuan Liu, Xuemei Wang, Tao Song, Aiai Song, Yibo Xi, Ting Zhang, Zilong Sun, Wei Li, Sheng Niu, Farwa Zainab, Chenyang Wang, Ding Zhang, Haidong Wang, Bo Yang

**Affiliations:** ^1^College of Veterinary Medicine, Shanxi Agricultural University, Jinzhong, China; ^2^College of Animal Science and Technology, Hebei Normal University of Science and Technology, Qinhuangdao, China; ^3^Xianyang Regional Wen's Animal Husbandry Co., Ltd., Xianyang, China; ^4^School of Management Shanxi Medical University, Taiyuan, China

**Keywords:** PDCoV, Huh7 cells, phylogenetic tree, transcriptome analysis, immune response

There was a mistake in Figure 9 as published. The bands representing the PDCoV N protein in interferon-related pathway and autophagy-related pathway were the same. This error occurred while we rearranged Figure 9D in the final version by selecting the same band of PDCoV N twice.

The corrected figure and its caption appear below.

**Figure 9:**
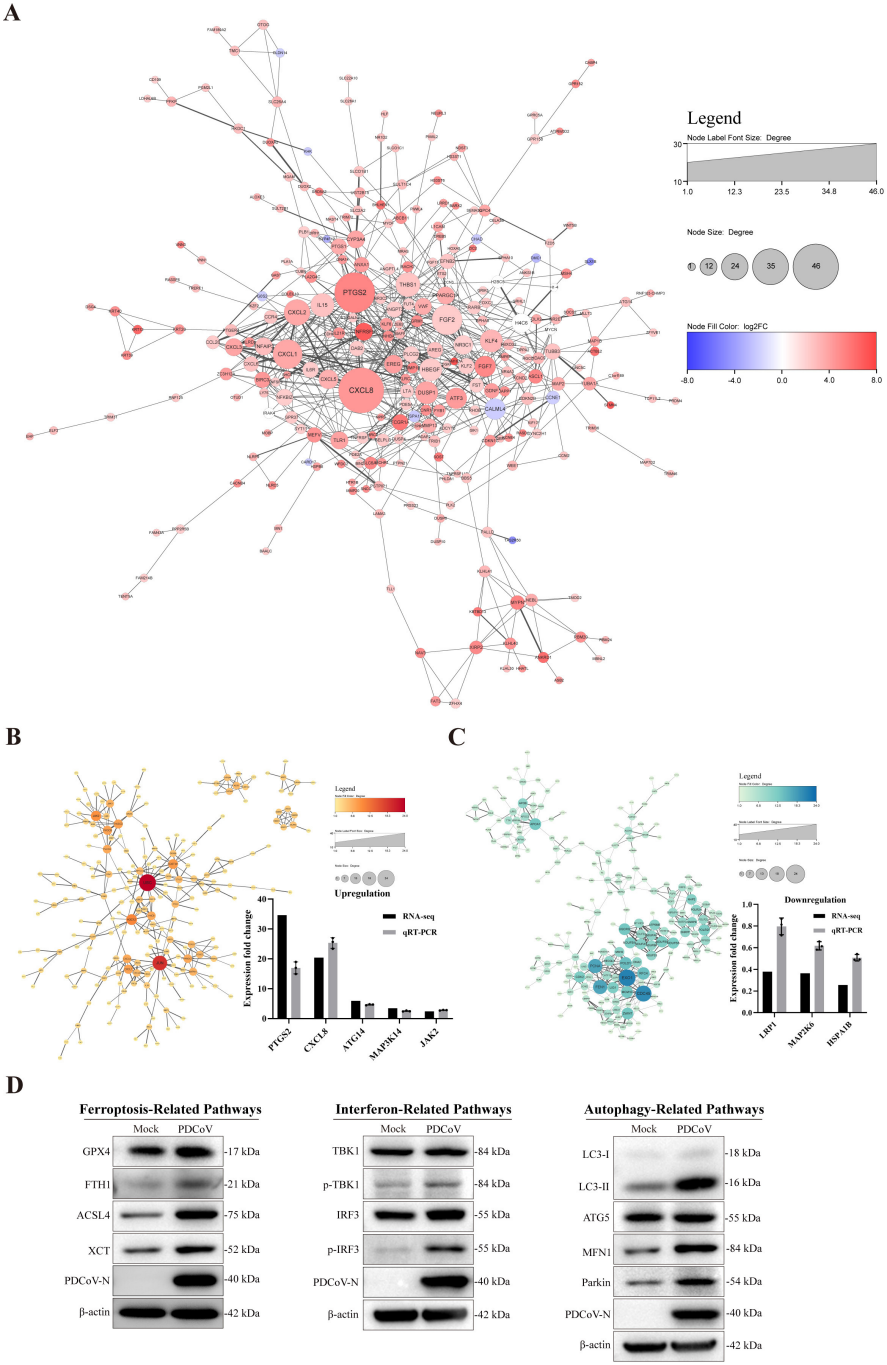
Interacting network of differentially expressed proteins. **(A)** PPI network of the DEGs. **(B, C)** PPI network of up-regulated and down-regulated DEGs and RT-qPCR verified results. **(D)** The related protein expression level of ferroptosis, autophagy and immune response in the context of PDCoV infection using western blotting. β-actin was used as an internal reference. All PPI networks were based on STRING analysis. Each node represented a protein, and each edge represented the interaction between proteins. The upregulated proteins are shown in red shadow, and the downregulated proteins are shown in blue.

The original version of this article has been updated.

