# Supplementary figures and images for: Isolation, phylogenetics, and characterization of a new PDCoV strain that affects cellular gene expression in human cells
Source: Front Microbiol. 2025 Mar 26;16:1534907. doi: 10.3389/fmicb.2025.1534907 (PMC11979167; doi:10.3389/fmicb.2025.1534907)

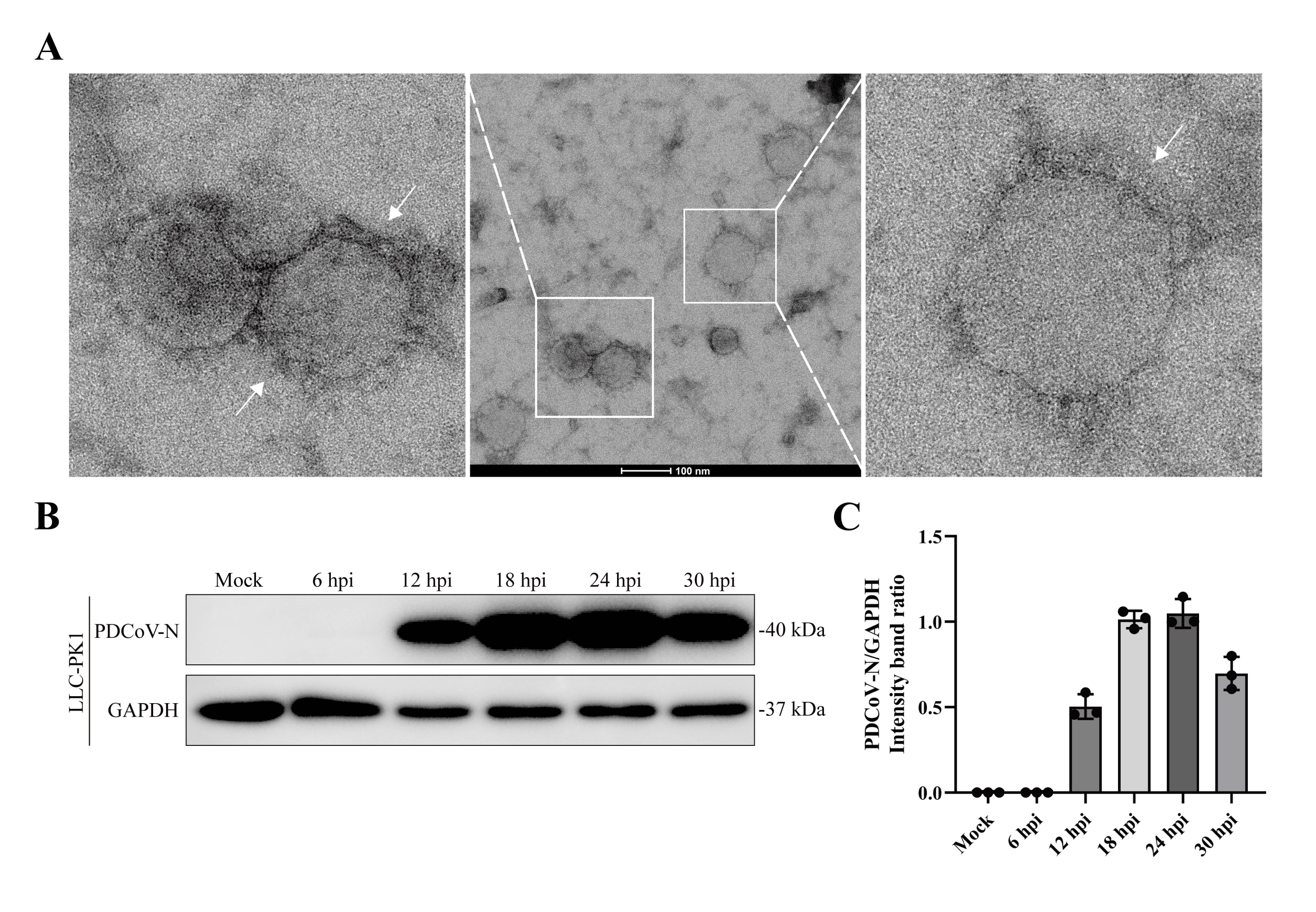

Supplement: Supplementary file 1 [file Image_1.jpg]

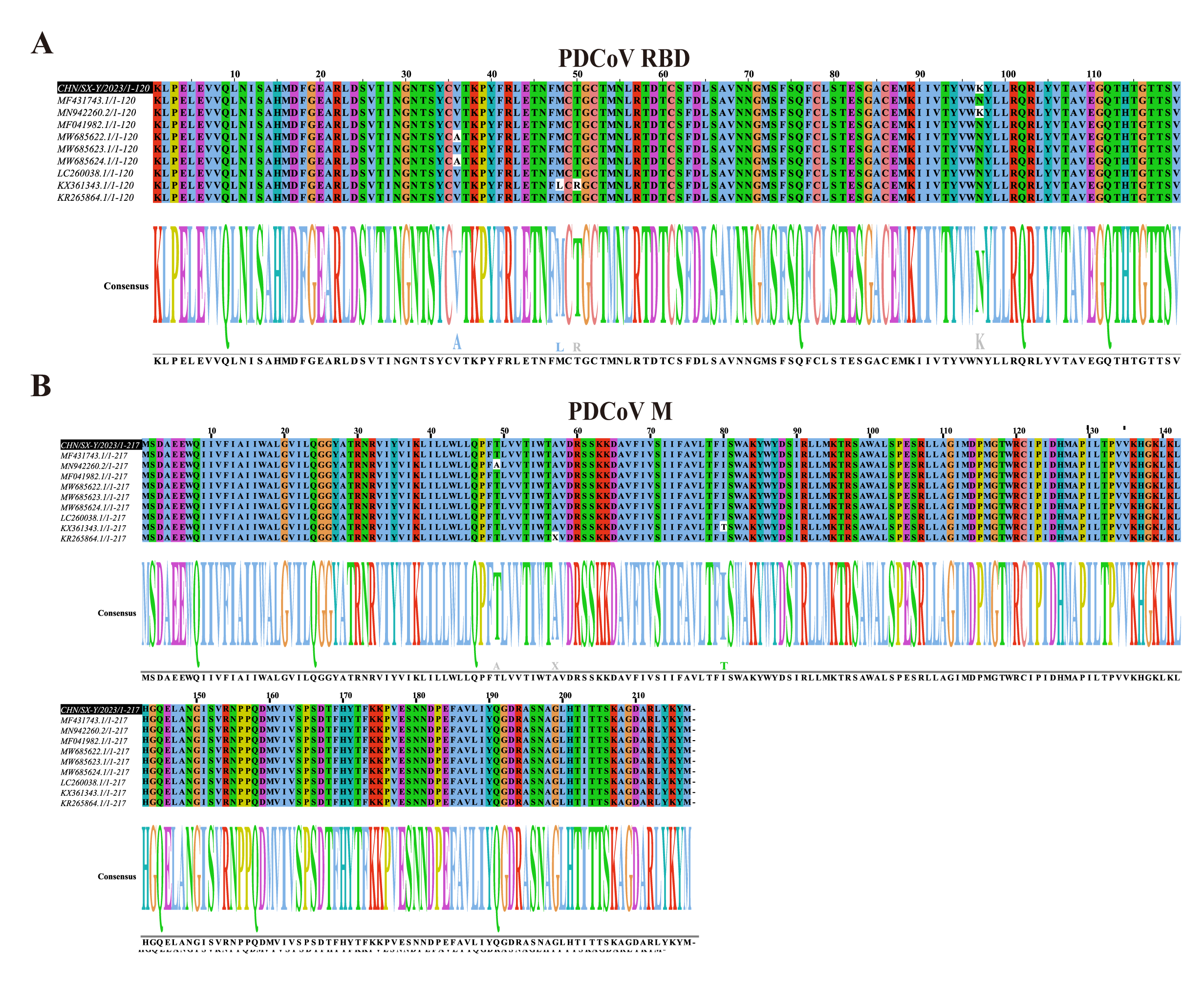

Supplement: Supplementary file 2 [file Image_2.TIF]

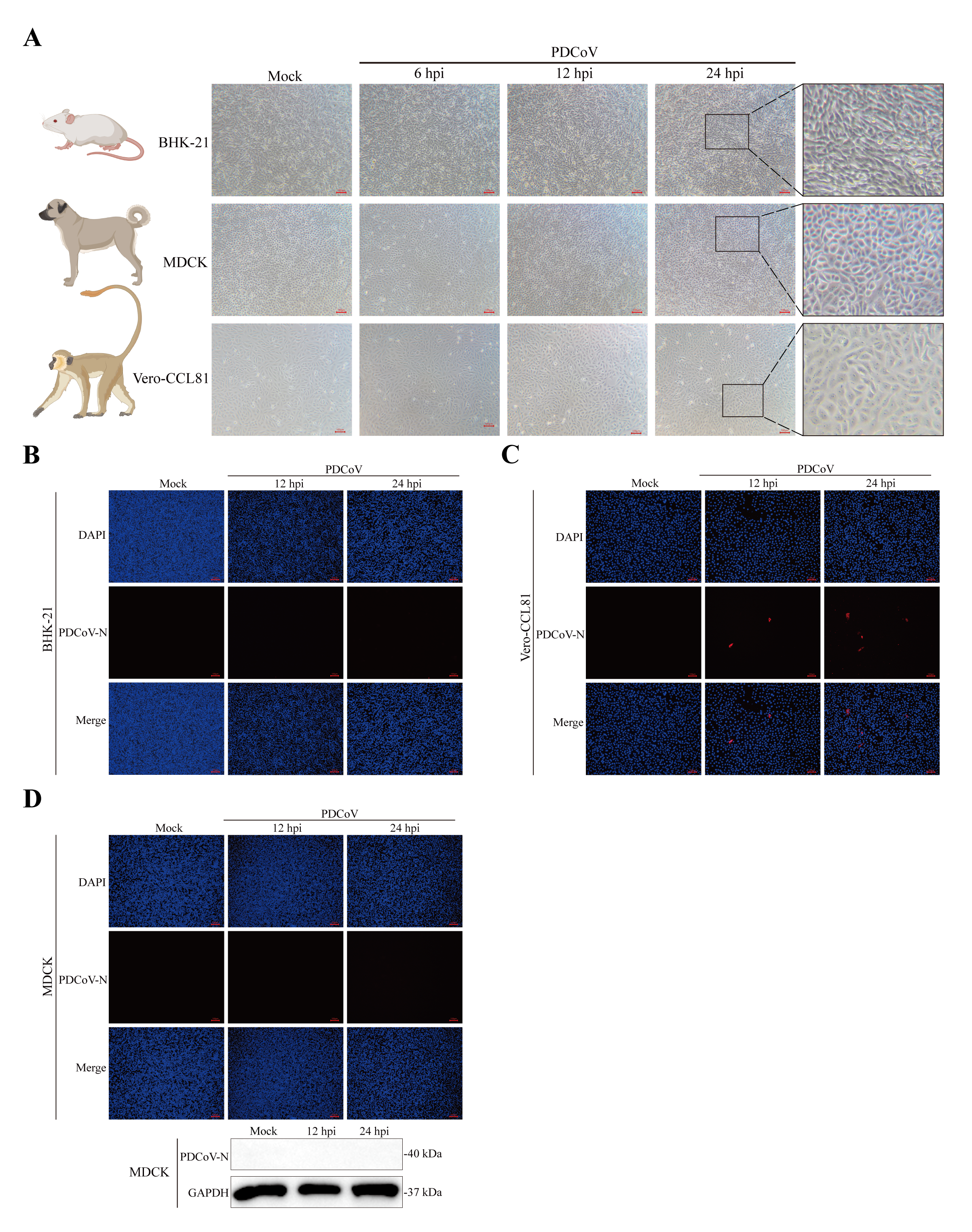

Supplement: Supplementary file 3 [file Image_3.TIF]
