# Supplementary material for: Isolation, phylogenetics, and characterization of a new PDCoV strain that affects cellular gene expression in human cells
Source: Front Microbiol. 2025 Mar 26;16:1534907. doi: 10.3389/fmicb.2025.1534907 (PMC11979167; doi:10.3389/fmicb.2025.1534907)
Supplement: Supplementary file 4 [file Table_1.DOCX]

**Supplementary Information**

**Figure legend**

Figure S1 The characterization of PDCoV CHN/SX-Y/2023. **(A)** Electron microscopic images of purified virus particles in LLC-PK1 cells. Bar, 100 nm. The white arrows represent coronary spike protein outline. **(B. C)** LLC-PK1 cells was infected with PDCoV (MOI = 1) and the expression levels of PDCoV N and GAPDH at different time points were detected using western blotting. Statistical significance is determined by *t* test (*P<0.05, **P<0.01).

Figure S2. Multiple sequence alignment based on the PDCoV RBD **(A)** and M **(B)** proteins of PDCoV strains. Partially identical nucleotides are combined and the white boxes represent different nucleotides. The black box represents PDCoV CHN/SX-Y/2023 strain.

Figure S3. The different cell susceptibility of PDCoV. **(A)** Cytopathic changes of different cell lines mock and infected with PDCoV CHN/SX-Y/2023 (MOI= 1) at 6, 12 and 24 hpi. Bar, 100 nm. **(B. C)** Immunofluorescence of PDCoV detected in mock and infected BHK-21 and Vero-CCL81 (MOI=1) at 12 and 24 hpi. **(D)** Immunofluorescence and western blot assay of PDCoV detected in mock and infected MDCK (MOI=1) at 12 and 24 hpi. The PDCoV N was detected to prove the existence and infection of the virus. Nuclei was strained blue with DAPI. GAPDH was as the internal reference. All images were taken at x10 magnification.

**Tables**

Table S1 The information of cell lines from the different species used in this study

| **Cell Name** | **Organism** | **Morphology** | **Tissue** |
| --- | --- | --- | --- |
| LLC-PK1 | Sus scrofa, pig | Epithelial | Kidney |
| ST | Sus scrofa, pig | Fibroblast | Testis |
| Vero CCL81 | Cercopithecus aethiops | Epithelial | Kidney |
| HuH-7 | Homo sapiens, human | Epithelial -Like | Liver, gallbladder |
| HEK-293T | Homo sapiens, human | Epithelial | Embryonic kidney |
| LMH | Gallus gallus, chicken | Epithelial | Liver |
| EEC | Capra hircus, goat | Epithelial | Endometria |
| MDBK | Bos taurus, cow | Epithelial | Kidney |
| MDCK | Canis familiaris, dog | Epithelial | Kidney |
| BHK21 | Mesocricetus auratus, hamster, Syrian golden | Fibroblast | Kidney |

Table S2 Oligonucleotide primers used for amplification of the complete genome of PDCoV CHN/SX-Y/2023 strain by RT-PCR

| **Primer** | **Sequence (5’-3’)** | **Nucleotide position** | **Product size (bp)** |
| --- | --- | --- | --- |
| PDCoV-1F  PDCoV-1R | ATGGGGACTAAAGATAAAAATTA  TCACAGTTGGAGATACTTCA | 1-1594 | 1594 |
| PDCoV-2F  PDCoV-2R | CTTCAGCAATTCTTGAACCG  TCAGAATCAAACTCCCCTTC | 1220-3050 | 1830 |
| PDCoV-3F  PDCoV-3R | TGTTGAACAACCAACCGAAACAGC  TTGGGTCATATTCACGAGCT | 2914-4412 | 1499 |
| PDCoV-4F  PDCoV-4R | TAGTAACTCTGACGCAGCAT  AGCTTCTGTCGGGATGAAAG | 4314-5733 | 1420 |
| PDCoV-5F  PDCoV-5R | TCGTTCAAGGCAGGAAATAC  CTCGACGTGCGGAAGCACAT | 5606-7155 | 1550 |
| PDCoV-6F  PDCoV-6R | TAGGCAAGCGTGTTGTAGTA  ATTAAGTTGGAGGACCACGT | 6968-8547 | 1580 |
| PDCoV-7F  PDCoV-7R | TGATGGCATTGTACGTCATG  AATGTTACGGTTTCCACCCA | 8412-9795 | 1384 |
| PDCoV-8F  PDCoV-8R | CGTTTGAGGTGCTTATAGCC  ATCCAGGACCCATAGTAGCA | 9737-11153 | 1417 |
| PDCoV-9F  PDCoV-9R | CTGTAGACCCTAAGACAGCC  TGACTGTTTAGCAATTCCGC | 11057-12623 | 1567 |
| PDCoV-10F  PDCoV-10R | AACTCCTTGTCAGACCTTGG  ACCTTGGTGAGGGGATATGC | 12572-14002 | 1431 |
| PDCoV-11F  PDCoV-11R | CTACTTACCTTACCCAGATG  AAACCAAACGAGCATTCACA | 13868-15354 | 1487 |
| PDCoV-12F  PDCoV-12R | ACCAATTATGAGCTTTCCTC  CATTAGTGAAACAAGCCCGC | 15315-16632 | 1318 |
| PDCoV-13F  PDCoV-13R | TTTGTCACTTGGGCTCACCA  GATTGTTATAGCGTACATAG | 16527-18030 | 1504 |
| PDCoV-14F  PDCoV-14R | GCAGTATTCCTCACCAAAAC  ATGTAAGAAGCGATGTGCAC | 17910-19429 | 1520 |
| PDCoV-15F  PDCoV-15R | TTGTGAGGCCACCAGAATGC  GATAGCACCGACAACGGTGT | 19306-20821 | 1516 |
| PDCoV-16F  PDCoV-16R | CCTCTCCAACGGGTGAGCTT  GCCTCTACTTCCTCAAGACG | 20699-21891 | 1193 |
| PDCoV-17F  PDCoV-17R | CAAACAATTTCCAAGCAATT  GTCAACTGCGGTCCAAATGG | 21825-23215 | 1391 |
| PDCoV-18F  PDCoV-18R | AAACTTATTCTGCTTTGGCT  GGATTGTTGGGGTTGCGTTT | 23150-24351 | 1202 |
| PDCoV-19F  PDCoV-19R | TCCTAATGATACCCCAGCAA  GCTCCATCCCCCCTATAAG | 24250-25414 | 1165 |

PDCoV, porcine deltacoronavirus.

Nucleotide position is numbered based on the PDCoV-HeN/swine/2015 strain (MN942260.2)

Table S3 154 PDCoV strains used for sequence alignment and phylogenetic analysis in this study

| **Accession** | **GenBank_Title** | **Release**  **Date** | **Collection**  **Date** | **Country** | **Length** |
| --- | --- | --- | --- | --- | --- |
| PQ373831 | Porcine deltacoronavirus isolate CHN/SX-Y/2023, complete genome | 2024.10.21 |  | China | 25415 |
| PP130097 | Porcine deltacoronavirus isolate HD2023, complete genome | 2024/1/24 |  | China | 25402 |
| OR762053 | Porcine deltacoronavirus isolate ZD2022, complete genome | 2023/11/13 | Jun-22 | China | 25410 |
| OR230676 | Porcine deltacoronavirus isolate HeN17, complete genome | 2023/9/20 | 2017 | China | 25420 |
| OP501870 | Porcine deltacoronavirus isolate CHN-HeN06-2022, complete genome | 2023/9/15 | 2022/5/10 | China | 25403 |
| OQ790128 | Porcine deltacoronavirus isolate PDCoV-KQ-Swine-2023, complete genome | 2023/8/8 | 2023/2/15 | China | 25402 |
| OQ790129 | Porcine deltacoronavirus isolate PDCoV-SN-Swine-2018, complete genome | 2023/8/8 | 2018/11/3 | China | 25389 |
| OR269935 | Porcine deltacoronavirus isolate PDCoV/WH/2023, complete genome | 2023/7/26 | 2023/5/15 | China | 25402 |
| OQ473581 | Porcine deltacoronavirus strain JS2021-LX, complete genome | 2023/7/18 | 2022 | China | 25435 |
| OQ736717 | Porcine deltacoronavirus isolate PDCoV-GX2022-1, complete genome | 2023/4/19 | 2021 | China | 25360 |
| OQ736716 | Porcine deltacoronavirus isolate PDCoV-GX2022-2, complete genome | 2023/4/19 | 2022 | China | 25355 |
| OQ547740 | Porcine deltacoronavirus isolate PDCoV-GX2021-1, complete genome | 2023/3/14 | 2021 | China | 25422 |
| OM047182 | Porcine deltacoronavirus isolate CHN-SCMY2021-01, complete genome | 2022/11/26 | 2021/4/2 | China | 25381 |
| OP566509 | Porcine deltacoronavirus isolate vPDCoV YRQ/2016, complete genome | 2022/11/9 | 2016 | China | 25418 |
| MZ388469 | Porcine deltacoronavirus isolate CH/GX/PDCoV/1423/2016, complete genome | 2022/8/15 | 2016/12/19 | China | 25393 |
| MZ388470 | Porcine deltacoronavirus isolate CH/GX/PDCoV/1472A/2017, complete genome | 2022/8/15 | 2017/1/22 | China | 25405 |
| MZ388471 | Porcine deltacoronavirus isolate CH/GX/PDCoV/1491B/2017, complete genome | 2022/8/15 | 2017/2/13 | China | 25405 |
| MZ388472 | Porcine deltacoronavirus isolate CH/GX/PDCoV/1539C/2017, complete genome | 2022/8/15 | 2017/3/3 | China | 25405 |
| MZ388473 | Porcine deltacoronavirus isolate CH/GX/PDCoV/1988A/2018, complete genome | 2022/8/15 | 2018/2/6 | China | 25382 |
| MZ388474 | Porcine deltacoronavirus isolate CH/GX/PDCoV/2081/2018, complete genome | 2022/8/15 | 2018/4/3 | China | 25398 |
| ON968724 | Porcine deltacoronavirus isolate CH/LNFX/2022, complete genome | 2022/8/15 | 2022/3/20 | China | 25506 |
| ON494594 | Porcine deltacoronavirus isolate CHN-SF-2018, complete genome | 2022/8/14 | 2018/2/15 | China | 25389 |
| MW816149 | Porcine deltacoronavirus strain CH7328, complete genome | 2022/7/5 | 2018 | China | 25420 |
| OL900393 | Porcine deltacoronavirus strain CHN-SCMY2021-02, complete genome | 2022/7/5 | 2021/4/2 | China | 25340 |
| OM777140 | Porcine deltacoronavirus strain NTU/C253/21, complete genome | 2022/6/11 | 2021 | Taiwan | 25392 |
| OM256446 | Porcine deltacoronavirus strain PDCoV-CH-SDLY52-2021, complete genome | 2022/2/27 | Nov-21 | China | 25403 |
| MZ772936 | Porcine deltacoronavirus isolate BN, complete genome | 2022/2/17 | 2016 | China | 25416 |
| OK546242 | Porcine deltacoronavirus strain CZ2020, complete genome | 2022/1/7 | 2020 | China | 25430 |
| MT663769 | Porcine deltacoronavirus strain CHN-TS1-2019, complete genome | 2021/9/8 | 2019/7/23 | China | 25420 |
| MZ802955 | Porcine deltacoronavirus isolate CH-HLJ-20, complete genome | 2021/8/22 | Sep-20 | China | 25403 |
| MW854634 | Porcine deltacoronavirus isolate 104-553, complete genome | 2021/8/8 | Jun-15 | Taiwan | 25418 |
| MT260150 | Porcine deltacoronavirus isolate HNZK-02-P15, complete genome | 2021/5/5 | 2018/6/5 | China | 25453 |
| MT260149 | Porcine deltacoronavirus isolate HNZK-02-P5, complete genome | 2021/5/5 | 2018/5/25 | China | 25520 |
| MK625638 | Porcine deltacoronavirus isolate CH/JXJGS01/2016, complete genome | 2021/1/1 | Oct-18 | China | 25447 |
| MK625639 | Porcine deltacoronavirus isolate CH/JXJGS01/2016, complete genome | 2021/1/1 | Oct-18 | China | 25438 |
| MK625640 | Porcine deltacoronavirus isolate CH/JXJGS01/2016, complete genome | 2021/1/1 | Oct-18 | China | 25447 |
| MK625641 | Porcine deltacoronavirus isolate CH/JXJGS01/2016, complete genome | 2021/1/1 | Oct-18 | China | 25446 |
| MN942260 | Porcine deltacoronavirus isolate PDcov HeN/swine/2015, complete genome | 2020/10/27 | 2015 | China | 25418 |
| MN781985 | Porcine deltacoronavirus isolate CHzmd2019, complete genome | 2020/9/22 |  | China | 25420 |
| MK359104 | Porcine deltacoronavirus isolate CHN-GX01-2018, complete genome | 2020/7/22 | 2018 | China | 25406 |
| MN173782 | Porcine deltacoronavirus isolate CHN-GX09-2018, complete genome | 2020/7/15 | 2018 | China | 25406 |
| MN173779 | Porcine deltacoronavirus isolate CHN-GX11-2018, complete genome | 2020/7/15 | 2018 | China | 25406 |
| MN173780 | Porcine deltacoronavirus isolate CHN-GX12-2018, complete genome | 2020/7/15 | 2018 | China | 25406 |
| MN173781 | Porcine deltacoronavirus isolate CHN-GX81-2018, complete genome | 2020/7/15 | 2018 | China | 25408 |
| MH025763 | Porcine deltacoronavirus strain CH/JXJGS01/P20, complete genome | 2020/3/6 | 2016 | China | 25438 |
| MH025764 | Porcine deltacoronavirus strain CH/JXJGS01/P50, complete genome | 2020/3/6 | 2016 | China | 25438 |
| MH025762 | Porcine deltacoronavirus strain CH/JXJGS01/P7, complete genome | 2020/3/6 | 2016 | China | 25438 |
| MK993519 | Porcine deltacoronavirus isolate CHN/Sichuan/2019, complete genome | 2019/12/24 | Jan-19 | China | 25380 |
| MN249445 | Porcine deltacoronavirus isolate CHN-JS-2017, complete genome | 2019/11/4 | 2017/12/11 | China | 25424 |
| MK330604 | Porcine deltacoronavirus strain CHN/Sichuan/2017, complete genome | 2019/7/27 | Feb-17 | China | 25402 |
| MK330605 | Porcine deltacoronavirus strain CHN/Sichuan/2018, complete genome | 2019/7/27 | Jan-18 | China | 25413 |
| MK355396 | Porcine deltacoronavirus strain CHN-SC2015, complete genome | 2019/7/8 | 2016/2/20 | China | 25403 |
| MK572803 | Porcine deltacoronavirus strain SCNC201705, complete genome | 2019/7/8 | Jun-17 | China | 25403 |
| MK211169 | Porcine deltacoronavirus strain CHN/Sichuan/2017, complete genome | 2019/6/30 | 2017/12/24 | China | 25393 |
| MK005882 | Porcine deltacoronavirus strain Swine/CHN/SC/2018/1, complete genome | 2019/5/14 | Mar-18 | China | 25414 |
| MH708123 | Porcine deltacoronavirus strain HNZK-02, complete genome | 2018/8/27 | 2018/3/20 | China | 25453 |
| MH708124 | Porcine deltacoronavirus strain HNZK-04, complete genome | 2018/8/27 | 2018/3/20 | China | 25454 |
| MH708125 | Porcine deltacoronavirus strain HNZK-06, complete genome | 2018/8/27 | 2018/3/20 | China | 25453 |
| KY293678 | Porcine deltacoronavirus isolate CH/JXJGS02/2016, complete genome | 2018/7/31 | 2016/5/23 | China | 25438 |
| MF642322 | Porcine deltacoronavirus strain CHN/GS/2016/1, complete genome | 2018/6/25 | Aug-16 | China | 25420 |
| MF642323 | Porcine deltacoronavirus strain CHN/GS/2016/2, complete genome | 2018/6/25 | Aug-16 | China | 25420 |
| MF642324 | Porcine deltacoronavirus strain CHN/GS/2017/1, complete genome | 2018/6/25 | Apr-17 | China | 25420 |
| MF642325 | Porcine deltacoronavirus strain CHN/QH/2017/1, complete genome | 2018/6/25 | Mar-17 | China | 25420 |
| MG242062 | Porcine deltacoronavirus isolate CHN-HeB1-2017, complete genome | 2018/4/22 | 2017 | China | 25414 |
| MF280390 | Porcine deltacoronavirus strain CHN-GD-2016, complete genome | 2018/4/2 | 2016 | China | 25402 |
| MF041982 | Porcine deltacoronavirus strain SHJS/SL/2016, complete genome | 2017/12/27 | 2016/12/23 | China | 25414 |
| KY513724 | Porcine deltacoronavirus strain CH/Hunan/2014, complete genome | 2017/12/26 | 2014 | China | 25413 |
| KY513725 | Porcine deltacoronavirus strain CH/Jiangsu/2014, complete genome | 2017/12/26 | 2014 | China | 25422 |
| KY363867 | Porcine deltacoronavirus isolate CHN-GD16-03, complete genome | 2017/11/20 | 2016/3/18 | China | 25396 |
| KY363868 | Porcine deltacoronavirus isolate CHN-GD16-05, complete genome | 2017/11/20 | 2016/1/5 | China | 25403 |
| MF948005 | Porcine deltacoronavirus strain HB-BD, complete sequence | 2017/10/16 | 2017/8/10 | China | 25423 |
| MF095123 | Porcine deltacoronavirus isolate CHN-HG-2017, complete genome | 2017/9/16 | 2017/2/15 | China | 25399 |
| MF431742 | Porcine deltacoronavirus strain GD, complete genome | 2017/8/9 | 2015 | China | 25420 |
| MF431743 | Porcine deltacoronavirus strain SD, complete genome | 2017/8/9 | 2014 | China | 25414 |
| KY293677 | Porcine deltacoronavirus isolate CH/JXJGS01/2016, complete genome | 2017/7/18 | 2016/5/23 | China | 25438 |
| KX443143 | Porcine deltacoronavirus strain CH-01, complete genome | 2017/2/27 | 2016 | China | 25404 |
| KY065120 | Porcine deltacoronavirus strain CHN/Tianjin/2016, complete genome | 2016/12/7 | 2016 | China | 25413 |
| JQ065043 | Porcine coronavirus HKU15 strain HKU15-155, complete genome | 2016/5/13 | 2010 | China: Hong Kong | 25425 |
| JQ065042 | Porcine coronavirus HKU15 strain HKU15-44, complete genome | 2016/5/13 | 2009 | China: Hong Kong | 25430 |
| KU981060 | Porcine deltacoronavirus strain NH isolate passage 0, complete genome | 2016/5/1 | 2015/4/15 | China | 25420 |
| KU981062 | Porcine deltacoronavirus strain NH isolate passage 10, complete genome | 2016/5/1 | 2015/12/30 | China | 25420 |
| KU981061 | Porcine deltacoronavirus strain NH isolate passage 5, complete genome | 2016/5/1 | 2015/7/18 | China | 25420 |
| KU981059 | Porcine deltacoronavirus strain NH, complete genome | 2016/5/1 | 2015/2/16 | China | 25420 |
| KT336560 | Porcine deltacoronavirus isolate CHN-HN-2014, complete genome | 2015/12/19 | 2014/11/24 | China | 25420 |
| KT021234 | Porcine deltacoronavirus strain CH/SXD1/2015, complete genome | 2015/9/16 | 2015/3/20 | China | 25419 |
| KR131621 | Porcine deltacoronavirus isolate PDCoV/CHJXNI2/2015, complete genome | 2015/8/25 | Mar-15 | China | 25438 |
| KT266822 | Porcine deltacoronavirus strain CH/Sichuan/S27/2012, complete genome | 2015/7/21 | 2012 | China | 25404 |
| KP757890 | Porcine deltacoronavirus isolate CHN-AH-2004, complete genome | 2015/4/20 | 2004/5/24 | China | 25420 |
| KP757891 | Porcine deltacoronavirus isolate CHN-HB-2014, complete genome | 2015/4/20 | 2014/12/26 | China | 25420 |
| KP757892 | Porcine deltacoronavirus isolate CHN-JS-2014, complete genome | 2015/4/20 | 2014/12/20 | China | 25420 |
| OQ718500 | Porcine deltacoronavirus isolate KPDCoV-2201 ORF1ab polyprotein (ORF1ab), ORF1a polyprotein (ORF1ab), spike protein (S), envelope protein (E), membrane protein (M), nonstructural protein (NS6), nucleocapsid protein (N), and nonstructural protein (NS7) genes, complete cds | 2023/6/12 | 2022 | South Korea | 25422 |
| OQ566226 | Porcine deltacoronavirus isolate GNU-2105, complete genome | 2023/3/20 | 2021 | South Korea | 25422 |
| OQ566227 | Porcine deltacoronavirus isolate GNU-2105-P1, complete genome | 2023/3/20 | 2021 | South Korea | 25422 |
| OQ566228 | Porcine deltacoronavirus isolate GNU-2105-P5, complete genome | 2023/3/20 | 2021 | South Korea | 25422 |
| MG837131 | Porcine deltacoronavirus isolate KNU16-07-P10, complete genome | 2018/5/7 | Nov-16 | South Korea | 25422 |
| MG837130 | Porcine deltacoronavirus isolate KNU16-07-P5, complete genome | 2018/5/7 | Nov-16 | South Korea | 25422 |
| KY926512 | Porcine deltacoronavirus isolate KNU16-11, complete genome | 2017/8/13 | Nov-16 | South Korea | 25419 |
| KY364365 | Porcine deltacoronavirus isolate KNU16-07, complete genome | 2017/4/24 | Jul-14 | South Korea | 25422 |
| KY354363 | Porcine deltacoronavirus isolate DH1, complete genome | 2017/3/4 | 2016/4/1 | South Korea | 25422 |
| KY354364 | Porcine deltacoronavirus isolate DH2, complete genome | 2017/3/4 | 2016/4/1 | South Korea | 25422 |
| LC260038 | Porcine deltacoronavirus genomic RNA, complete genome, strain: AKT/JPN/2014 | 2018/4/11 | May-14 | Japan | 25362 |
| LC260039 | Porcine deltacoronavirus genomic RNA, complete genome, strain: GNM-1/JPN/2014 | 2018/4/11 | May-14 | Japan | 25362 |
| LC260040 | Porcine deltacoronavirus genomic RNA, complete genome, strain: GNM-2/JPN/2014 | 2018/4/11 | May-14 | Japan | 25362 |
| LC260041 | Porcine deltacoronavirus genomic RNA, complete genome, strain: IWT/JPN/2014 | 2018/4/11 | May-14 | Japan | 25362 |
| LC260042 | Porcine deltacoronavirus genomic RNA, complete genome, strain: MYZ/JPN/2014 | 2018/4/11 | Mar-14 | Japan | 25362 |
| LC260043 | Porcine deltacoronavirus genomic RNA, complete genome, strain: OKN/JPN/2014 | 2018/4/11 | Aug-14 | Japan | 25362 |
| LC260044 | Porcine deltacoronavirus genomic RNA, complete genome, strain: YMG/JPN/2014 | 2018/4/11 | Dec-14 | Japan | 25362 |
| LC260045 | Porcine deltacoronavirus genomic RNA, complete genome, strain: HKD/JPN/2016 | 2017/6/29 | Sep-16 | Japan | 25359 |
| MZ802772 | Porcine deltacoronavirus strain PDCoV/CBR-1/2016/Thailand, complete genome | 2022/1/10 | Sep-16 | Thailand | 25406 |
| MZ802773 | Porcine deltacoronavirus strain PDCoV/CBR-2/2016/Thailand, complete genome | 2022/1/10 | Sep-16 | Thailand | 25406 |
| MZ802774 | Porcine deltacoronavirus strain PDCoV/CBR-3/2016/Thailand, complete genome | 2022/1/10 | Sep-16 | Thailand | 25406 |
| MZ802775 | Porcine deltacoronavirus strain PDCoV/NKP-1/2016/Thailand, complete genome | 2022/1/10 | Jan-16 | Thailand | 25406 |
| MZ802777 | Porcine deltacoronavirus strain PDCoV/RBR-1/2016/Thailand, complete genome | 2022/1/10 | May-16 | Thailand | 25406 |
| KX361343 | Porcine deltacoronavirus isolate P1_13_ST1_0213/PDCoV/0213/Thailand, complete genome | 2020/5/31 | Feb-13 | Thailand | 25406 |
| KX361345 | Porcine deltacoronavirus isolate P24_15_NT1_1215/PDCoV/2015/Thailand, complete genome | 2020/5/31 | Dec-15 | Thailand | 25406 |
| KX361344 | Porcine deltacoronavirus isolate P2_13_ST2_0313/PDCoV/0213/Thailand, complete genome | 2020/5/31 | Mar-13 | Thailand | 25406 |
| KU984334 | Porcine deltacoronavirus isolate TT_1115, complete genome | 2016/4/12 | Nov-15 | Thailand | 25403 |
| KU051641 | Porcine deltacoronavirus strain PDCoV/Swine/Thailand/S5011/2015, complete genome | 2016/3/9 | 2015/6/10 | Thailand | 25405 |
| KU051649 | Porcine deltacoronavirus strain PDCoV/Swine/Thailand/S5015L/2015, complete genome | 2016/3/9 | 2015/6/30 | Thailand | 25405 |
| MZ802776 | Porcine deltacoronavirus strain PDCoV/VUT-1/2016/Vietnam, complete genome | 2022/1/10 | Mar-16 | Viet Nam | 25406 |
| KX998969 | Porcine deltacoronavirus isolate P29_15_VN_1215, complete genome | 2020/1/1 | Dec-15 | Viet Nam | 25415 |
| KX834352 | Porcine deltacoronavirus strain PDCoV/Swine/Vietnam/Binh21/2015, complete genome | 2017/2/6 | 2015/12/8 | Viet Nam | 25406 |
| KX834351 | Porcine deltacoronavirus strain PDCoV/Swine/Vietnam/HaNoi6/2015, complete genome | 2017/2/6 | 2015/10/10 | Viet Nam | 25406 |
| KX118627 | Porcine deltacoronavirus isolate P1_16_BTL_0115/PDCoV/2016/Lao, complete genome | 2016/9/13 | 2016/1/20 | Laos | 25405 |
| KX022602 | Porcine deltacoronavirus strain PDCoV/USA/Iowa136/2015, complete genome | 2016/6/11 | 2015/10/15 | USA | 25382 |
| KX022603 | Porcine deltacoronavirus strain PDCoV/USA/Minnesota140/2015, complete genome | 2016/6/11 | 2015/12/18 | USA | 25394 |
| KX022604 | Porcine deltacoronavirus strain PDCoV/USA/Nebraska137/2015, complete genome | 2016/6/11 | 2015/11/27 | USA | 25382 |
| KX022605 | Porcine deltacoronavirus strain PDCoV/USA/Nebraska145/2015, complete genome | 2016/6/11 | 2015/12/21 | USA | 25320 |
| MZ291567 | Porcine deltacoronavirus strain OH-FD22 P7, complete genome | 2021/6/28 | 2014 | USA | 25438 |
| MW196362 | Mutant Porcine deltacoronavirus strain USA/IL/2014/026PDV_P11, complete genome | 2020/12/2 | 2019 | USA | 25447 |
| KR150443 | Porcine deltacoronavirus strain USA/Arkansas61/2015, complete genome | 2016/2/29 | 2015/3/24 | USA | 25398 |
| KR265856 | Porcine deltacoronavirus strain USA/Illinois272/2014, complete genome | 2016/2/29 | 2014/2/23 | USA | 25399 |
| KR265857 | Porcine deltacoronavirus strain USA/Illinois273/2014, complete genome | 2016/2/29 | 2014/2/23 | USA | 25394 |
| KR265852 | Porcine deltacoronavirus strain USA/Illinois449/2014, complete genome | 2016/2/29 | 2014/4/21 | USA | 25394 |
| KR265851 | Porcine deltacoronavirus strain USA/Indiana453/2014, complete genome | 2016/2/29 | 2014/5/13 | USA | 25394 |
| KR265865 | Porcine deltacoronavirus strain USA/Iowa459/2014, complete genome | 2016/2/29 | 2014/6/5 | USA | 25394 |
| KR265849 | Porcine deltacoronavirus strain USA/Michigan447/2014, complete genome | 2016/2/29 | 2014/4/2 | USA | 25393 |
| KR265850 | Porcine deltacoronavirus strain USA/Michigan448/2014, complete genome | 2016/2/29 | 2014/4/2 | USA | 25394 |
| KR265853 | Porcine deltacoronavirus strain USA/Minnesota/2013, complete genome | 2016/2/29 | 2013/10/14 | USA | 25394 |
| KR265859 | Porcine deltacoronavirus strain USA/Minnesota159/2014, complete genome | 2016/2/29 | 2014/2/11 | USA | 25401 |
| KR265848 | Porcine deltacoronavirus strain USA/Minnesota214/2014, complete genome | 2016/2/29 | 2014/3/14 | USA | 25396 |
| KR265864 | Porcine deltacoronavirus strain USA/Minnesota292/2014, complete genome | 2016/2/29 | 2014/3/14 | USA | 25395 |
| KR265847 | Porcine deltacoronavirus strain USA/Minnesota442/2014, complete genome | 2016/2/29 | 2014/3/6 | USA | 25394 |
| KR265854 | Porcine deltacoronavirus strain USA/Minnesota454/2014, complete genome | 2016/2/29 | 2014/5/21 | USA | 25394 |
| KR265855 | Porcine deltacoronavirus strain USA/Minnesota455/2014, complete genome | 2016/2/29 | 2014/5/21 | USA | 25394 |
| KR265860 | Porcine deltacoronavirus strain USA/Nebraska209/2014, complete genome | 2016/2/29 | 2014/2/5 | USA | 25396 |
| KR265861 | Porcine deltacoronavirus strain USA/Nebraska210/2014, complete genome | 2016/2/29 | 2014/2/5 | USA | 25404 |
| KR265858 | Porcine deltacoronavirus strain USA/NorthCarolina452/2014, complete genome | 2016/2/29 | 2014/5/6 | USA | 25394 |
| KR265862 | Porcine deltacoronavirus strain USA/Ohio444/2014, complete genome | 2016/2/29 | 2014/3/26 | USA | 25394 |
| KR265863 | Porcine deltacoronavirus strain USA/Ohio445/2014, complete genome | 2016/2/29 | 2014/3/27 | USA | 25394 |
| KP981395 | Porcine deltacoronavirus strain USA/IL/2014/026PDV_P11, complete genome | 2015/4/8 |  | USA | 25422 |
| MW685622 | Porcine deltacoronavirus isolate PDCoV/Haiti/Human/0081-4/2014, complete genome | 2021/6/9 | 2014/12/15 | Haiti | 25444 |
| MW685623 | Porcine deltacoronavirus isolate PDCoV/Haiti/Human/0256-1/2015, complete genome | 2021/6/9 | 2015/3/16 | Haiti | 25447 |
| MW685624 | Porcine deltacoronavirus isolate PDCoV/Haiti/Human/0329-4/2015, complete genome | 2021/6/9 | 2015/4/13 | Haiti | 25444 |

PDCoV, porcine deltacoronavirus.

Table S4 Coding potential and putative transcription regulatory sequences of the PDCoV CHN/SX-Y/2023 genome

| **ORF** | **Location (nt)** | **Length (nt)** | **Length (aa)** |
| --- | --- | --- | --- |
| 1ab | 540-19339 | 18796 | 6265 |
| S | 19318-22797 | 3480 | 1160 |
| E | 22791-23042 | 252 | 84 |
| M | 23035-23688 | 654 | 218 |
| NS6 | 23688-23972 | 285 | 95 |
| N | 23993-25021 | 1029 | 343 |

aa: amino acid

Table S5 Amino acid sequence similarity analysis between PDCoV CHN/SX-Y/2023 (PQ373831) and the reference strains

| **strains** | **PQ373831**  **S** | **PQ373831**  **RBD** | **PQ373831**  **M** | **PQ373831**  **N** |
| --- | --- | --- | --- | --- |
| MF431743.1 | 99.2 | 99.2 | 100 | 100 |
| MN942260.2 | 99.7 | 100 | 99.5 | 100 |
| MF041982.1 | 98.7 | 99.2 | 100 | 99.4 |
| MW685622.1 | 98.0 | 98.3 | 100 | 99.1 |
| MW685623.1 | 98.3 | 99.2 | 100 | 99.4 |
| MW685624.1 | 98.1 | 98.3 | 100 | 99.1 |
| LC260038.1 | 98.4 | 99.2 | 100 | 99.4 |
| KX361343.1 | 96.4 | 97.5 | 99.5 | 98.8 |
| KR265864.1 | 98.3 | 99.2 | 99.5 | 99.1 |

Table S6 Statistical analysis of sequencing data

| **Sample** | **Raw reads** | **Raw bases** | **Clean reads** | **Clean bases** | **Q20** | **Q30** | **GC%** |
| --- | --- | --- | --- | --- | --- | --- | --- |
| Mock1 | 45303088 | 6.80G | 44020708 | 6.60G | 98.61 | 96.19 | 47.96 |
| Mock 2 | 44498528 | 6.67G | 43104724 | 6.47G | 98.58 | 96.06 | 48.37 |
| Mock 3 | 48035074 | 7.21G | 46212046 | 6.93G | 99.12 | 97.51 | 48.99 |
| PDCoV1 | 45592722 | 6.84G | 44017910 | 6.60G | 98.66 | 96.32 | 47.38 |
| PDCoV2 | 44314552 | 6.65G | 42930390 | 6.44G | 98.55 | 95.97 | 47.87 |
| PDCoV3 | 47020372 | 7.05G | 46169088 | 6.93G | 98.63 | 96.27 | 46.76 |

Mock: Huh7 cells PDCoV: Huh7 cells infected PDCoV

Q20: The percentage of bases with Phred values greater than 20 in total bases

Q30: The percentage of bases with Phred values greater than 30 in total bases

GC%: The percentage of GC in total bases

Table S7 Top 50 of differentially expressed genes following mock and PDCoV infected Huh7 cells

| **Gene_id** | **Gene_name** | **Log2 FoldChange** | **Pvalue** | **Gene_description** |
| --- | --- | --- | --- | --- |
| ENSG00000154319 | FAM167A | 7.300266024 | 1.85E-08 | family with sequence similarity 167 member A [Source:HGNC Symbol;Acc:HGNC:15549] |
| ENSG00000073737 | DHRS9 | 7.235506284 | 2.28E-08 | dehydrogenase/reductase 9 [Source:HGNC Symbol;Acc:HGNC:16888] |
| ENSG00000157103 | SLC6A1 | 7.063518653 | 9.51E-08 | solute carrier family 6 member 1 [Source:HGNC Symbol;Acc:HGNC:11042] |
| ENSG00000189325 | C6orf222 | 6.931255368 | 7.66E-38 | chromosome 6 open reading frame 222 [Source:HGNC Symbol;Acc:HGNC:33769] |
| ENSG00000015592 | STMN4 | 6.776988807 | 8.05E-08 | stathmin 4 [Source:HGNC Symbol;Acc:HGNC:16078] |
| ENSG00000049249 | TNFRSF9 | 6.743984374 | 4.66E-07 | TNF receptor superfamily member 9 [Source:HGNC Symbol;Acc:HGNC:11924] |
| ENSG00000225913 | AL138767.3 | 6.742143062 | 5.57E-07 | novel transcript |
| ENSG00000231298 | MANCR | 6.524232782 | 2.39E-06 | mitotically associated long non coding RNA [Source:HGNC Symbol;Acc:HGNC:44678] |
| ENSG00000101842 | VSIG1 | 6.465983846 | 3.09E-06 | V-set and immunoglobulin domain containing 1 [Source:HGNC Symbol;Acc:HGNC:28675] |
| ENSG00000253227 | AC090192.2 | 6.46289694 | 2.57E-32 | novel transcript |
| ENSG00000187720 | THSD4 | 6.415668948 | 7.09E-57 | thrombospondin type 1 domain containing 4 [Source:HGNC Symbol;Acc:HGNC:25835] |
| ENSG00000188460 | ACTBP11 | 6.3819006 | 3.79E-06 | actin, beta pseudogene 11 [Source:HGNC Symbol;Acc:HGNC:33971] |
| ENSG00000187242 | KRT12 | 6.337384889 | 5.74E-06 | keratin 12 [Source:HGNC Symbol;Acc:HGNC:6414] |
| ENSG00000233397 | AC008063.2 | 6.146715133 | 1.65E-05 | novel transcript |
| ENSG00000148677 | ANKRD1 | 6.053074647 | 1.65E-61 | ankyrin repeat domain 1 [Source:HGNC Symbol;Acc:HGNC:15819] |
| ENSG00000162520 | SYNC | 6.051726942 | 3.90E-06 | syncoilin, intermediate filament protein [Source:HGNC Symbol;Acc:HGNC:28897] |
| ENSG00000203985 | LDLRAD1 | 6.041826949 | 5.64E-06 | low density lipoprotein receptor class A domain containing 1 [Source:HGNC Symbol;Acc:HGNC:32069] |
| ENSG00000169067 | ACTBL2 | 5.942667847 | 6.95E-05 | actin, beta like 2 [Source:HGNC Symbol;Acc:HGNC:17780] |
| ENSG00000234913 | AC016027.2 | 5.898486498 | 5.35E-05 | novel transcript |
| ENSG00000253270 | AC090541.1 | 5.798845556 | 8.51E-05 | novel transcript |
| ENSG00000113600 | C9 | 5.783552489 | 1.43E-05 | complement C9 [Source:HGNC Symbol;Acc:HGNC:1358] |
| ENSG00000182836 | PLCXD3 | 5.773095347 | 2.51E-07 | phosphatidylinositol specific phospholipase C X domain containing 3 [Source:HGNC Symbol;Acc:HGNC:31822] |
| ENSG00000272347 | AC116351.2 | 5.739139526 | 0.000141842 | novel transcript |
| ENSG00000123095 | BHLHE41 | 5.718591965 | 4.03E-07 | basic helix-loop-helix family member e41 [Source:HGNC Symbol;Acc:HGNC:16617] |
| ENSG00000150337 | FCGR1A | 5.695032978 | 0.000140804 | Fc fragment of IgG receptor Ia [Source:HGNC Symbol;Acc:HGNC:3613] |
| ENSG00000168746 | LINC01620 | 5.675581172 | 0.000163603 | long intergenic non-protein coding RNA 1620 [Source:HGNC Symbol;Acc:HGNC:16195] |
| ENSG00000177409 | SAMD9L | 5.640193185 | 0.00018484 | sterile alpha motif domain containing 9 like [Source:HGNC Symbol;Acc:HGNC:1349] |
| ENSG00000227621 | PHBP11 | 5.626381266 | 0.000234824 | prohibitin pseudogene 11 [Source:HGNC Symbol;Acc:HGNC:39290] |
| ENSG00000056291 | NPFFR2 | 5.585886448 | 1.58E-09 | neuropeptide FF receptor 2 [Source:HGNC Symbol;Acc:HGNC:4525] |
| ENSG00000167941 | SOST | 5.582303269 | 0.000310431 | sclerostin [Source:HGNC Symbol;Acc:HGNC:13771] |
| ENSG00000257302 | FAHD2P1 | 5.575050216 | 1.02E-06 | fumarylacetoacetate hydrolase domain containing 2 pseudogene 1 [Source:HGNC Symbol;Acc:HGNC:32441] |
| ENSG00000234438 | KBTBD13 | 5.5582102 | 0.000294296 | kelch repeat and BTB domain containing 13 [Source:HGNC Symbol;Acc:HGNC:37227] |
| ENSG00000254119 | AC025524.2 | 5.529245518 | 0.000413508 | uncharacterized LOC101929628 [Source:NCBI gene;Acc:101929628] |
| ENSG00000223553 | SMPD4P1 | 5.520457737 | 0.00047249 | sphingomyelin phosphodiesterase 4 pseudogene 1 [Source:HGNC Symbol;Acc:HGNC:39673] |
| ENSG00000261089 | CDC37P2 | 5.451312361 | 0.000465035 | cell division cycle 37 pseudogene 2 [Source:HGNC Symbol;Acc:HGNC:35453] |
| ENSG00000271824 | SMIM32 | 5.43242826 | 0.000510402 | small integral membrane protein 32 [Source:HGNC Symbol;Acc:HGNC:53640] |
| ENSG00000043355 | ZIC2 | 5.369443729 | 0.000137683 | Zic family member 2 [Source:HGNC Symbol;Acc:HGNC:12873] |
| ENSG00000258455 | AL158801.3 | 5.323329961 | 0.000794806 | novel transcript |
| ENSG00000261349 | AL031432.2 | -5.310818545 | 0.000613244 | novel pseudogene |
| ENSG00000255874 | LINC00346 | 5.310298191 | 0.000745308 | long intergenic non-protein coding RNA 346 [Source:HGNC Symbol;Acc:HGNC:27492] |
| ENSG00000236173 | AL049612.1 | -5.284199946 | 0.000124415 | novel transcript |
| ENSG00000213943 | KRT18P17 | 5.232062325 | 0.001173491 | keratin 18 pseudogene 17 [Source:HGNC Symbol;Acc:HGNC:33385] |
| ENSG00000276116 | FUT8-AS1 | 5.169982701 | 0.001549864 | FUT8 antisense RNA 1 [Source:HGNC Symbol;Acc:HGNC:44294] |
| ENSG00000205670 | SMIM11A | -5.160154196 | 6.56E-14 | small integral membrane protein 11A [Source:HGNC Symbol;Acc:HGNC:1293] |
| ENSG00000138347 | MYPN | 5.15405166 | 0.001457081 | myopalladin [Source:HGNC Symbol;Acc:HGNC:23246] |
| ENSG00000108551 | RASD1 | 5.146323928 | 5.67E-100 | ras related dexamethasone induced 1 [Source:HGNC Symbol;Acc:HGNC:15828] |
| ENSG00000073756 | PTGS2 | 5.113218287 | 0.000307841 | prostaglandin-endoperoxide synthase 2 [Source:HGNC Symbol;Acc:HGNC:9605] |
| ENSG00000093134 | VNN3 | 5.102588197 | 4.79E-146 | vanin 3 [Source:HGNC Symbol;Acc:HGNC:16431] |
| ENSG00000139352 | ASCL1 | 5.083172011 | 0.002840621 | achaete-scute family bHLH transcription factor 1 [Source:HGNC Symbol;Acc:HGNC:738] |
| ENSG00000267311 | AC007673.1 | 5.082413337 | 0.001940952 | novel transcript, sense intronic to RAB27B |

Table S8 Top 10 of GO analysis in BP, CC and MF in PDCoV infected Huh7 cells

| **Category** | **GO ID** | **Description** | **Gene Ratio** | **P value** | **Gene Name** |
| --- | --- | --- | --- | --- | --- |
| BP | GO:0048514 | blood vessel morphogenesis | 94/1710 | 1.21E-06 | ANXA1/ZC3H12A/HDAC9/RHOB/FZD5 et al. |
| BP | GO:0006935 | chemotaxis | 91/1710 | 4.68E-06 | ANXA1/CXCL5/CXCL1/THBS1/IL6R et al. |
| BP | GO:0042330 | taxis | 91/1710 | 5.10E-06 | ANXA1/CXCL5/CXCL1/THBS1/IL6R et al. |
| BP | GO:0040017 | positive regulation of locomotion | 80/1710 | 5.30E-05 | ANXA1/CXCL5/ZC3H12A/HDAC9/RHOB et al. |
| BP | GO:0001525 | angiogenesis | 77/1710 | 4.38E-05 | ANXA1/ZC3H12A/HDAC9/RHOB/FZD5 et al. |
| BP | GO:0051272 | positive regulation of cellular component movement | 75/1710 | 0.000206 | ANXA1/CXCL5/ZC3H12A/HDAC9/RHOB et al. |
| BP | GO:2000147 | positive regulation of cell motility | 75/1710 | 9.63E-05 | ANXA1/CXCL5/ZC3H12A/HDAC9/RHOB et al. |
| BP | GO:0030335 | positive regulation of cell migration | 71/1710 | 0.000238 | ANXA1/CXCL5/ZC3H12A/HDAC9/RHOB et al. |
| BP | GO:0060537 | muscle tissue development | 62/1710 | 9.43E-05 | ATF3/HDAC9/BMP2/NR1D2/MAFF et al. |
| BP | GO:0014706 | striated muscle tissue development | 59/1710 | 0.000150 | ATF3/HDAC9/BMP2/NR1D2/MAFF et al. |
| CC | GO:0031012 | extracellular matrix | 65/1797 | 0.001287 | THBS1/LAMA3/TNFRSF11B/CTGF/THSD4 et al. |
| CC | GO:0005578 | proteinaceous extracellular matrix | 53/1797 | 0.001825 | LAMA3/TNFRSF11B/CTGF/THSD4/DST et al. |
| CC | GO:0045177 | apical part of cell | 49/1797 | 0.043948 | ANXA1/PTPRH/IL6R/MUC13/DSTYK et al. |
| CC | GO:0016324 | apical plasma membrane | 42/1797 | 0.027697 | ANXA1/PTPRH/IL6R/MUC13/DSTYK et al. |
| CC | GO:0031225 | anchored component of membrane | 22/1797 | 0.024296 | VNN3/RAB27B/CD109/ART4/VNN2 et al. |
| CC | GO:0044420 | extracellular matrix component | 18/1797 | 0.043034 | LAMA3/THSD4/DST/NTN4/NID1 et al. |
| CC | GO:0042734 | presynaptic membrane | 15/1797 | 0.01191 | STX3/NLGN2/SYP/SYT11/SYT1 et al. |
| CC | GO:0005604 | basement membrane | 15/1797 | 0.027591 | LAMA3/DST/NTN4/NID1/LAMB3 et al. |
| CC | GO:0031463 | Cul3-RING ubiquitin ligase complex | 14/1797 | 0.014044 | BACH1/BACH2/SPOPL/TNFAIP1/KLHL24 et al. |
| CC | GO:0005881 | cytoplasmic microtubule | 13/1797 | 0.01657 | TUBG1/CLIP1/TUBA1A/HID1/GRAMD2B et al. |
| MF | GO:0004842 | ubiquitin-protein transferase activity | 67/1732 | 0.005912 | NEURL3/TNFAIP3/BIRC3/ITCH/NEDD4 et al. |
| MF | GO:0019787 | ubiquitin-like protein transferase activity | 67/1732 | 0.021405 | NEURL3/TNFAIP3/BIRC3/ITCH/NEDD4 et al. |
| MF | GO:0000987 | proximal promoter sequence-specific DNA binding | 66/1732 | 0.000927 | KLF6/ATF3/ELF3/ETS2/PRDM4 et al. |
| MF | GO:0000982 | transcription factor activity, RNA polymerase II proximal promoter sequence-specific DNA binding | 65/1732 | 0.000145 | KLF6/ATF3/ELF3/ETS2/PRDM4 et al. |
| MF | GO:0000978 | RNA polymerase II proximal promoter sequence-specific DNA binding | 63/1732 | 0.001979 | KLF6/ATF3/ELF3/ETS2/PRDM4 et al. |
| MF | GO:0001228 | transcriptional activator activity, RNA polymerase II transcription regulatory region sequence-specific DNA binding | 62/1732 | 0.000799 | KLF6/ATF3/ELF3/PRDM4/NR5A2 et al. |
| MF | GO:0030545 | receptor regulator activity | 59/1732 | 0.000986 | CXCL5/AREG/BMP2/CXCL1/TNFRSF11B et al. |
| MF | GO:0048018 | receptor ligand activity | 55/1732 | 0.001756 | CXCL5/AREG/BMP2/CXCL1/TNFRSF11B et al. |
| MF | GO:0042578 | phosphoric ester hydrolase activity | 52/1732 | 0.029092 | DUSP1/DUSP4/DUSP8/PHLPP2/PTPRH et al. |
| MF | GO:0001227 | transcriptional repressor activity, RNA polymerase II transcription regulatory region sequence-specific DNA binding | 40/1732 | 0.000425 | ATF3/ETS2/NR1D2/ZNF281/MAFK et al. |

Table S9 Top 15 gene sets of GSEA analysis of GO in PDCoV infected Huh7 cells

| Gene set name | GO ID | Size | ES | NES |
| --- | --- | --- | --- | --- |
| extrinsic component of organelle membrane | 0031312 | 46 | 0.53 | 2.67 |
| RNA polymerase II distal enhancer sequence_specific dna binding | 0000980 | 91 | 0.43 | 2.28 |
| cell cycle arrest | 0007050 | 223 | 0.35 | 2.25 |
| phosphatase activator activity | 0019211 | 16 | 0.54 | 2.22 |
| peptidyl_tyrosine dephosphorylation | 0035335 | 97 | 0.49 | 2.19 |
| phosphatase regulator activity | 0019208 | 91 | 0.40 | 2.19 |
| regulation of synaptic vesicle cycle | 0098693 | 64 | 0.48 | 2.19 |
| negative regulation of cyclin_dependent protein kinase activity | 1904030 | 31 | 0.44 | 2.18 |
| cellular response to alcohol | 0097306 | 54 | 0.47 | 2.18 |
| enhancer sequence_specific dna binding | 0001158 | 108 | 0.38 | 2.18 |
| enhancer binding | 0035326 | 122 | 0.40 | 2.17 |
| protein tyrosine phosphatase activity | 0004725 | 96 | 0.50 | 2.16 |
| phosphoric ester hydrolase activity | 0042578 | 349 | 0.35 | 2.16 |
| regulation of phosphatase activity | 0010921 | 159 | 0.37 | 2.16 |
| phosphatase activity | 0016791 | 257 | 0.37 | 2.15 |

Table S10 Top 20 gene sets of GSEA analysis of KEGG in PDCoV infected Huh7 cells

| Gene set name | ID | Size | ES | NES |
| --- | --- | --- | --- | --- |
| AMPK signaling pathway | HSA04152 | 112 | 0.39 | 2.06 |
| TGF_beta signaling pathway | HSA04350 | 75 | 0.36 | 2.01 |
| Hepatitis C | HSA05160 | 110 | 0.39 | 1.99 |
| pantothenate and coa biosynthesis | HSA00770 | 18 | 0.56 | 1.96 |
| ubiquitin mediated proteolysis | HSA04120 | 130 | 0.35 | 1.96 |
| measles | HSA05162 | 96 | 0.43 | 1.96 |
| melanoma | HSA05218 | 64 | 0.44 | 1.91 |
| rheumatoid arthritis | HSA05323 | 57 | 0.52 | 1.89 |
| EGFR tyrosine kinase inhibitor resistance | HSA01521 | 70 | 0.46 | 1.86 |
| phospholipase D signaling pathway | HSA04072 | 128 | 0.45 | 1.86 |
| endocytosis | HSA04144 | 221 | 0.32 | 1.85 |
| autophagy animal | HSA04140 | 122 | 0.47 | 1.84 |
| HIPPO signaling pathway | HSA04390 | 137 | 0.36 | 1.83 |
| Kaposi sarcoma associated herpesvirus infection | HSA05167 | 156 | 0.39 | 1.83 |
| FOXO signaling pathway | HSA04068 | 121 | 0.48 | 1.82 |

Table S11 Top 15 of the Reactome, Do and DisGeNET analysis in PDCoV infected Huh7 cells

| ID name | Description | Gene  Ratio | P value | Partial Gene name |
| --- | --- | --- | --- | --- |
| Reactome: |  |  |  |  |
| R-HSA-1474244 | Extracellular matrix organization | 44/1063 | 0.009181897 | BMP2/THBS1/LAMA3/SDC4/DST |
| R-HSA-983231 | Factors involved in megakaryocyte development and platelet production | 26/1063 | 0.017575065 | WEE1/MAFF/MAFK/GATA6/CABLES1 |
| R-HSA-1474228 | Degradation of the extracellular matrix | 23/1063 | 0.009469751 | LAMA3/CAPN2/NID1/LAMB3/CAPN7 |
| R-HSA-6785807 | Interleukin-4 and 13 signaling | 17/1063 | 0.025519386 | ANXA1/MCL1/IL6R/CXCL8/RHOU |
| R-HSA-1474290 | Collagen formation | 15/1063 | 0.039577421 | LAMA3/DST/LAMB3/TLL1/COL2A1 |
| R-HSA-877300 | Interferon gamma signaling | 14/1063 | 0.046964015 | TRIM31/JAK1/ICAM1/SP100/TRIM21 |
| R-HSA-383280 | Nuclear Receptor transcription pathway | 13/1063 | 0.002796473 | NR5A2/NR1D2/NR3C1/THRB/NR3C2 |
| R-HSA-6791312 | TP53 Regulates Transcription of Cell Cycle Genes | 13/1063 | 0.002796473 | PLK2/PCNA/BTG2/CNOT4/CDK2 |
| R-HSA-2022090 | Assembly of collagen fibrils and other multimeric structures | 13/1063 | 0.007006277 | LAMA3/DST/LAMB3/TLL1/COL2A1 |
| R-HSA-168643 | Nucleotide-binding domain, leucine rich repeat containing receptor (NLR) signaling pathways | 12/1063 | 0.008022746 | TNFAIP3/BIRC3/ITCH/IRAK2/MAP2K6 |
| R-HSA-112409 | RAF-independent MAPK1/3 activation | 11/1063 | 1.14E-05 | DUSP1/DUSP4/DUSP10/IL6R/JAK1 |
| R-HSA-6783783 | Interleukin-10 signaling | 11/1063 | 0.002054632 | CXCL1/CXCL8/CXCL2/IL18/JAK1 |
| R-HSA-380108 | Chemokine receptors bind chemokines | 11/1063 | 0.002619388 | CXCL5/CXCL1/CXCL8/CXCL2/CXCL3 |
| R-HSA-373752 | Netrin-1 signaling | 11/1063 | 0.01528841 | ABLIM3/NTN4/TRIO/WASL/SLIT2 |
| R-HSA-9012852 | Signaling by NOTCH3 | 11/1063 | 0.01528841 | WWC1/JAG1/NCSTN/KAT2A/PBX1 |
| DO: |  |  |  |  |
| DOID:10763 | hypertension | 99/816 | 2.02E-07 | BMP2/IL6R/JUN/TNFRSF11B/PDE5A |
| DOID:3093 | nervous system cancer | 81/816 | 0.003623 | CDKN2B/IL6R/JUN/SQSTM1/TGFBR2 |
| DOID:7148 | rheumatoid arthritis | 79/816 | 0.000297 | MCL1/IL6R/JUN/TNFRSF11B/CXCL8/NR3C1 |
| DOID:1579 | respiratory system disease | 75/816 | 0.001609 | CXCL5/AREG/JUN/TNFRSF11B/CXCL8 |
| DOID:114 | heart disease | 73/816 | 0.003587 | TNFRSF11B/PDE5A/CXCL8/HSPA1B/JAG1 |
| DOID:850 | lung disease | 69/816 | 0.000911 | ANXA1/CXCL5/AREG/TNFRSF11B/CXCL8 |
| DOID:0050161 | lower respiratory tract disease | 69/816 | 0.001498 | ANXA1/CXCL5/AREG/TNFRSF11B/CXCL8 |
| DOID:10591 | pre-eclampsia | 44/816 | 0.000889 | IL6R/TNFRSF11B/CXCL8/F2RL1/IL18 |
| DOID:2320 | obstructive lung disease | 43/816 | 0.005412 | AREG/TNFRSF11B/CXCL8/NR3C1/HSPA1B |
| DOID:8398 | osteoarthritis | 33/816 | 0.001149 | TNFAIP3/PTGER4/SDC4/IL6R/CXCL8 |
| DOID:403 | mouth disease | 31/816 | 0.001031 | CDKN2B/TNFRSF11B/CXCL8/F2RL1/IL18 |
| DOID:5295 | intestinal disease | 27/816 | 0.004914 | BIRC3/CXCL8/HSPA1B/IL18/CAB39 |
| DOID:1091 | tooth disease | 26/816 | 0.002464 | CDKN2B/TNFRSF11B/CXCL8/F2RL1/IL18 |
| DOID:3388 | periodontal disease | 25/816 | 0.000365 | TNFRSF11B/CXCL8/F2RL1/IL18/AGT |
| DOID:3620 | central nervous system cancer | 24/816 | 0.004059 | CDKN2B/PCNA/JAK1/RASSF1/GADD45A |
| DisGeNET: |  |  |  |  |
| C0011881 | Diabetic Nephropathy | 91/1468 | 8.52E-06 | KLF6/BMP2/THBS1/MLLT3/IL6R |
| C0021368 | Inflammation | 84/1468 | 9.89E-05 | ANXA1/ELF3/CXCL1/THBS1/PTGER4 |
| C0020456 | Hyperglycemia | 82/1468 | 1.09E-07 | CDKN2B/THBS1/IL6R/JUN/TNFRSF11B |
| C0151744 | Myocardial Ischemia | 80/1468 | 2.93E-07 | ATF3/DUSP1/ZC3H12A/AREG/TRIB1 |
| C0042373 | Vascular Diseases | 75/1468 | 1.06E-07 | AREG/CDKN2B/THBS1/JUN/TNFRSF11B |
| C0948008 | Ischemic stroke | 66/1468 | 4.50E-07 | ZC3H12A/HDAC9/CDKN2B/TRIB1/TNFRSF11B |
| C0278883 | Metastatic melanoma | 59/1468 | 1.36E-05 | KLF6/WEE1/PHLDA1/RHOB/CDKN2B |
| C3495559 | Juvenile arthritis | 56/1468 | 9.82E-07 | DUSP1/HDAC9/AREG/DUSP4/CXCL1 |
| C3272363 | Ischemic Cerebrovascular Accident | 55/1468 | 1.99E-05 | ZC3H12A/HDAC9/CDKN2B/TRIB1/TNFRSF11B |
| C3811653 | Experimental Organism Basal Cell Carcinoma | 48/1468 | 1.02E-05 | BMP2/CDKN2B/MCL1/JUN/CXCL8 |
| C0022876 | Premature Obstetric Labor | 46/1468 | 6.84E-05 | CXCL5/BMP2/THBS1/PTGER4/IL6R |
| C1704436 | Peripheral Arterial Diseases | 23/1468 | 9.70E-05 | ATF3/BMP2/THBS1/CXCL8/PCSK9 |
| C0879615 | Stromal Neoplasm | 21/1468 | 4.70E-05 | ATF3/CXCL5/MCL1/TNFRSF11B/CXCL8 |
| C0006271 | Bronchiolitis | 13/1468 | 0.000142 | JUN/CXCL8/NR3C1/IL18/VDR |
